# UFMylation of NLRP3 Prevents Its Autophagic Degradation and Facilitates Inflammasome Activation

**DOI:** 10.1002/advs.202406786

**Published:** 2025-02-22

**Authors:** Jiongjie Jing, Fan Yang, Ke Wang, Mintian Cui, Ni Kong, Shixi Wang, Xiaoyue Qiao, Fanyu Kong, Dongyang Zhao, Jinlu Ji, Lunxian Tang, Jiaxin Gao, Yu‐Sheng Cong, Deqiang Ding, Kun Chen

**Affiliations:** ^1^ State Key Laboratory of Cardiovascular Diseases and Medical Innovation Center Shanghai East Hospital School of Life Sciences and Technology Tongji University Shanghai 200127 China; ^2^ Shanghai Key Laboratory of Maternal Fetal Medicine Clinical and Translational Research Center of Shanghai First Maternity and Infant Hospital School of Life Sciences and Technology Tongji University Shanghai 200092 China; ^3^ Department of Internal Emergency Medicine Shanghai East Hospital School of Medicine Tongji University Shanghai 200120 China; ^4^ State Key Laboratory of Mycology Institute of Microbiology Chinese Academy of Sciences Beijing 100101 China; ^5^ Key Laboratory of Aging and Cancer Biology of Zhejiang Province Institute of Aging Research School of Medicine Hangzhou Normal University Hangzhou 311121 China; ^6^ Shanghai Key Laboratory of Signaling and Disease Research Frontier Science Center for Stem Cell Research School of Life Sciences and Technology Tongji University Shanghai 200092 China

**Keywords:** autophagic degradation, NLRP3 inflammasome, therapeutic target, UFMylation

## Abstract

NLRP3 (NOD, LRR and pyrin domain‐containing protein 3) inflammasome is important for host defense against infections and maintaining homeostasis. Aberrant activation of NLRP3 inflammasome is closely related to various inflammatory diseases. Post‐translational modifications are critical for NLRP3 inflammasome regulation. However, the mechanism of NLRP3 inflammasome activation remains incompletely understood. Here, it is demonstrated that the Ufm1 E3 ligase Ufl1 mediated UFMylation is essential for NLRP3 inflammasome activation. Mechanistically, Ufl1 binds and UFMylates NLRP3 in the priming stage of NLRP3 activation, thereby sustaining the stability of NLRP3 by preventing NLRP3 K63‐linked ubiquitination and the subsequent autophagic degradation. It is further demonstrated that myeloid cell‐specific *Ufl1* or *Ufm1* deficiency in mice significantly alleviated inflammatory responses and tissue damage following lipopolysaccharide (LPS)‐induced endotoxemia and alum‐induced peritonitis. Thus, the findings offer new insights into potential therapeutic targets for NLRP3 inflammasome‐related diseases by targeting the UFMylation system.

## Graphical abstract

This article studies the role of UFMylation in NLRP3 inflammasome activation. It reveals that UFMylation, mediated by the E3 Ufm1‐ligase 1 (Ufl1), maintains NLRP3 stability by preventing its ubiquitination and subsequent autophagic degradation. Myeloid cell‐specific deletion of *Ufl1* or ubiquitin‐fold modifier 1 (*Ufm1*) significantly alleviates inflammatory responses in LPS‐induced endotoxemia and alum‐induced peritonitis. This research provides new insights into the regulation of NLRP3 inflammasome activation and inflammation.

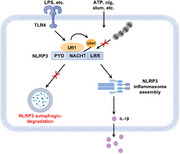

## 1 Introduction

Inflammasomes are large multiprotein complexes of the innate immune response that serve as platforms for caspase‐1 activation, interleukin‐1β (IL‐1β), and IL‐18 production, as well as inflammatory pyroptosis.^[^ ^1^, ^2^, ^3^ ^]^ The assembly of inflammasomes depends on cytosolic sensing of signals from microbial infections and endogenous danger signals by pattern recognition receptors (PRRs), such as nucleotide binding oligomerization domain (NOD)‐like receptors (NLRs) and absent in melanoma 2 (AIM2)‐like receptors.^[^ ^1^, ^4^ ^]^ NLRP3 inflammasome plays a crucial role in host defense against infections and maintaining homeostasis because NLRP3 can sense a diverse range of stimuli, including bacterial toxins, ATP, crystalline substances, and β‐amyloid peptide.^[^ ^5^, ^6^, ^7^ ^]^ Therefore, the activation of NLRP3 inflammasome must be precisely regulated to avoid deleterious pathological injuries. Aberrant activation of NLRP3 inflammasome is associated with various inflammatory diseases, including autoimmune diseases,^[^ ^8^ ^]^ cancer,^[^ ^9^ ^]^ metabolic diseases,^[^ ^5^ ^]^ cardiovascular dysfunction,^[^ ^5^ ^]^ neurological diseases,^[^ ^5^ ^]^ and COVID‐19.^[^ ^10^, ^11^ ^]^ Additionally, genetic mutations of NLRP3 which lead to excessive activation of the NLRP3 inflammasome contribute to cryopyrinopathies or cryopyrin‐associated periodic fever syndromes (CAPSs) and very‐early‐onset inflammatory bowel disease.^[^ ^12^, ^13^ ^]^ Therefore, uncovering the key regulatory mechanisms for NLRP3 inflammasome activation is vital for the development of potential therapeutic strategies for treating inflammatory diseases.

The activation of NLRP3 inflammasome is achieved by a two‐step process including priming and assembly in which NLRP3 protein level is a critical prerequisite for this inflammatory response.^[^ ^5^, ^14^ ^]^ During priming and assembly, NLRP3 is tightly regulated by multiple post‐translational modifications (PTMs), including ubiquitination, phosphorylation, and acetylation.^[^ ^6^, ^15^, ^16^, ^17^, ^18^ ^]^ In addition, some nonclassical PTMs, such as SUMOylation, nitrosylation, and palmitoylation of NLRP3 were identified in different phases of the NLRP3 inflammasome.^[^ ^19^, ^20^, ^21^ ^]^ Thus, NLRP3 inflammasome activity is controlled by dynamic landscape of PTMs, while whether there are new PTMs of NLRP3 and how different types of PTMs work in a cross‐talk manner remain elusive.

Protein UFMylation is a novel reversible PTM mediated by conjugating a ubiquitin‐like protein, ubiquitin‐fold modifier 1 (Ufm1) to proteins.^[^ ^22^ ^]^ Similar to ubiquitination, UFMylation modification requires an E1‐E2‐E3 cascade reaction after maturation of Ufm1 precursor cleaved by Ufm1 specific cysteine proteases (Ufsp1 and Ufsp2).^[^ ^23^ ^]^ Unlike the ubiquitination, only one of each enzyme has been discovered to mediate UFMylation till now.^[^ ^24^ ^]^ Uba5 works as the E1 activator to activate mature Ufm1 and transfer it to E2 Ufm1‐conjugase 1 (Ufc1). Finally, the E3 Ufm1‐ligase 1 (Ufl1), recruits the substrate for UFMylation.^[^ ^23^, ^25^ ^]^ Accumulating evidence displays that UFMylation is involved in regulation of diverse biological processes, including cellular differentiation,^[^ ^26^ ^]^ ER homeostasis,^[^ ^27^, ^28^, ^29^ ^]^ DNA damage response,^[^ ^30^, ^31^ ^]^ and cancer.^[^ ^32^, ^33^ ^]^ More recently, UFMylation has been reported to affect antiviral immunity by targeting several key antiviral factors, such as Retinoic acid‐inducible gene I (RIG‐I) and mitochondrial antiviral‐signaling protein (MAVS).^[^ ^34^, ^35^, ^36^, ^37^ ^]^ However, whether UFMylation regulates the key inflammatory component NLRP3 is unknown.

Herein, we show that Ufl1 interacts with and catalyzes NLRP3 for UFMylation. Furthermore, Ufl1‐mediated UFMylation occurs in the NACHT domain of NLRP3, which prevents ubiquitination of NLRP3 and subsequent autophagy‐mediated degradation, thereby sustaining the NLRP3 inflammasome activation. Deficiency of *Ufl1* or *Ufm1* in mice shows a significantly attenuated inflammatory response in LPS‐induced endotoxemia, alum‐induced peritonitis and *E. Coli*‐induced sepsis. Our findings suggest that targeting the UFMylation system would be a potential therapeutic treatment in control of inflammatory disorders.

## 2 Results

### 2.1 Ufl1 and Ufm1 are Down‐Regulated upon NLRP3 Inflammasome Activation

**Figure 1:**
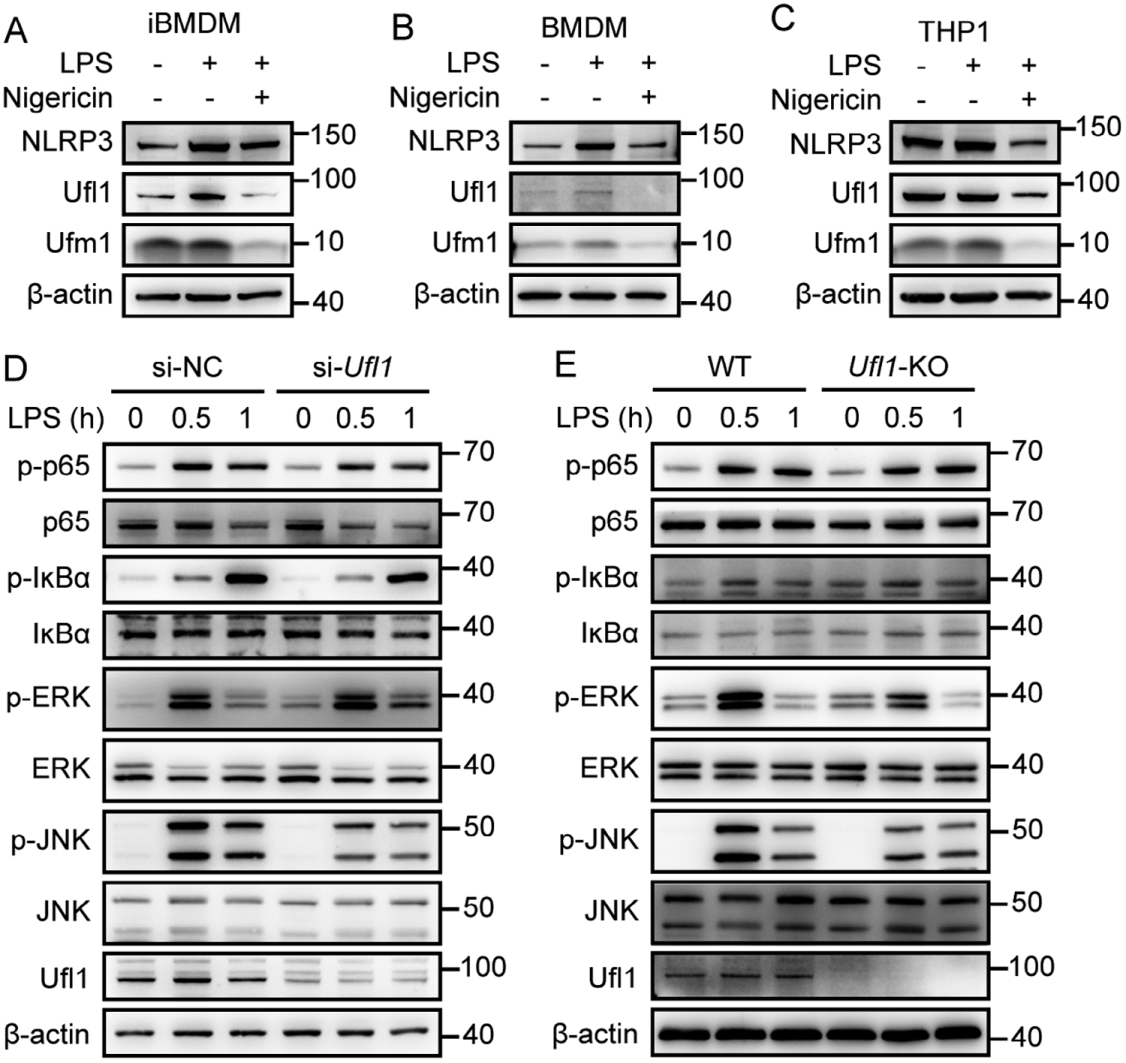
Core components in UFMylation cascade are down‐regulated upon NLRP3 inflammasome activation. A–C) Immunoblot analysis of NLRP3, Ufl1, and Ufm1 in the whole cell lysis of LPS‐stimulated (200 ng mL^−1^, 4 h) or LPS‐primed (200 ng mL^−1^, 4 h) and nigericin‐activated (10 µm, 1 h) iBMDMs A), BMDMs B), and THP1 C). D) Immunoblot analysis of p‐p65, p65, p‐IκBα, IκBα, p‐ERK, ERK, p‐JNK, and JNK in the whole cell lysis of LPS‐stimulated iBMDMs transfected with scramble sequence (si‐NC) or specific sequences targeting Ufl1 (si‐*Ufl1*). E) Immunoblot analysis of p‐p65, p65, p‐IκBα, IκBα, p‐ERK, ERK, p‐JNK, and JNK in the whole cell lysis of LPS‐stimulated WT and *Ufl1‐*KO BMDMs. Data are representative of three independent experiments.

To investigate the role of UFMylation in regulation of NLRP3 inflammasome activation, we first determined the expression of the core components (Ufl1, Ufm1, Ufc1, and DDRGK domain‐containing protein 1 (Ddrgk1)) in UFMylation cascade upon the NLRP3 inflammasome activation in immortalized bone marrow‐derived macrophages (iBMDMs), mouse BMDMs, and human myeloid leukemia mononuclear cells (THP‐1)‐derived macrophages. We found that most components, including Ufl1, Ufm1, and Ufc1, consistently downregulated in different types of macrophages (iBMDMs, BMDMs, and THP1‐derived macrophages) (**Figure** **1**A–C; and Figure S1A–C, Supporting Information). In addition, Ddrgk1 was also downregulated in BMDMs and THP1‐derived macrophages, and displayed no significant changes in iBMDMs. Uba5 showed decreased expression in THP1‐derived macrophages, but no significant changes in iBMDMs and BMDMs (Figure S1A–C, Supporting Information). These data indicate that Ufl1 and Ufm1, the key players in UFMylation, are closely related to NLRP3 inflammasome activation.

We then explored the role of UFMylation in NLRP3 inflammasome activation by knocking down the expression of *Ufl1* and *Ufm1*, the key components in UFMylation. First, we found that in priming stage of NLRP3 inflammasome, *Ufl1* and *Ufm1* silencing by small interfering RNAs (siRNAs) significantly decreased phosphorylation of ERK and JNK, but mildly influenced the NF‐κB pathway in LPS‐stimulated iBMDMs (Figure 1D; and Figure S1D, Supporting Information). We then generated mice with myeloid cell‐specific deletion of *Ufl1* or *Ufm1* by crossing *Ufl1*‐floxed or *Ufm1*‐floxed mice with *Lyz2*‐cre mice. Consistently, LPS‐primed *Ufl1* or *Ufm1*‐deficient BMDMs showed downregulated phosphorylation of ERK and JNK (Figure 1E; and Figure S1E, Supporting Information). In addition, the expression of *Il1b* and *Nlrp3* in *Ufl1*‐KO BMDMs was lower than that in WT BMDMs (Figure S1F,G, Supporting Information). Together, these results indicate that deficiency of *Ufl1* or *Ufm1*, the key components of UFMylation, could suppress LPS‐induced priming of NLRP3 inflammasome through downregulating MAPK signaling pathway.

### 2.2 *Ufl1* Deficiency Attenuates NLRP3 Inflammasome Activation

**Figure 2:**
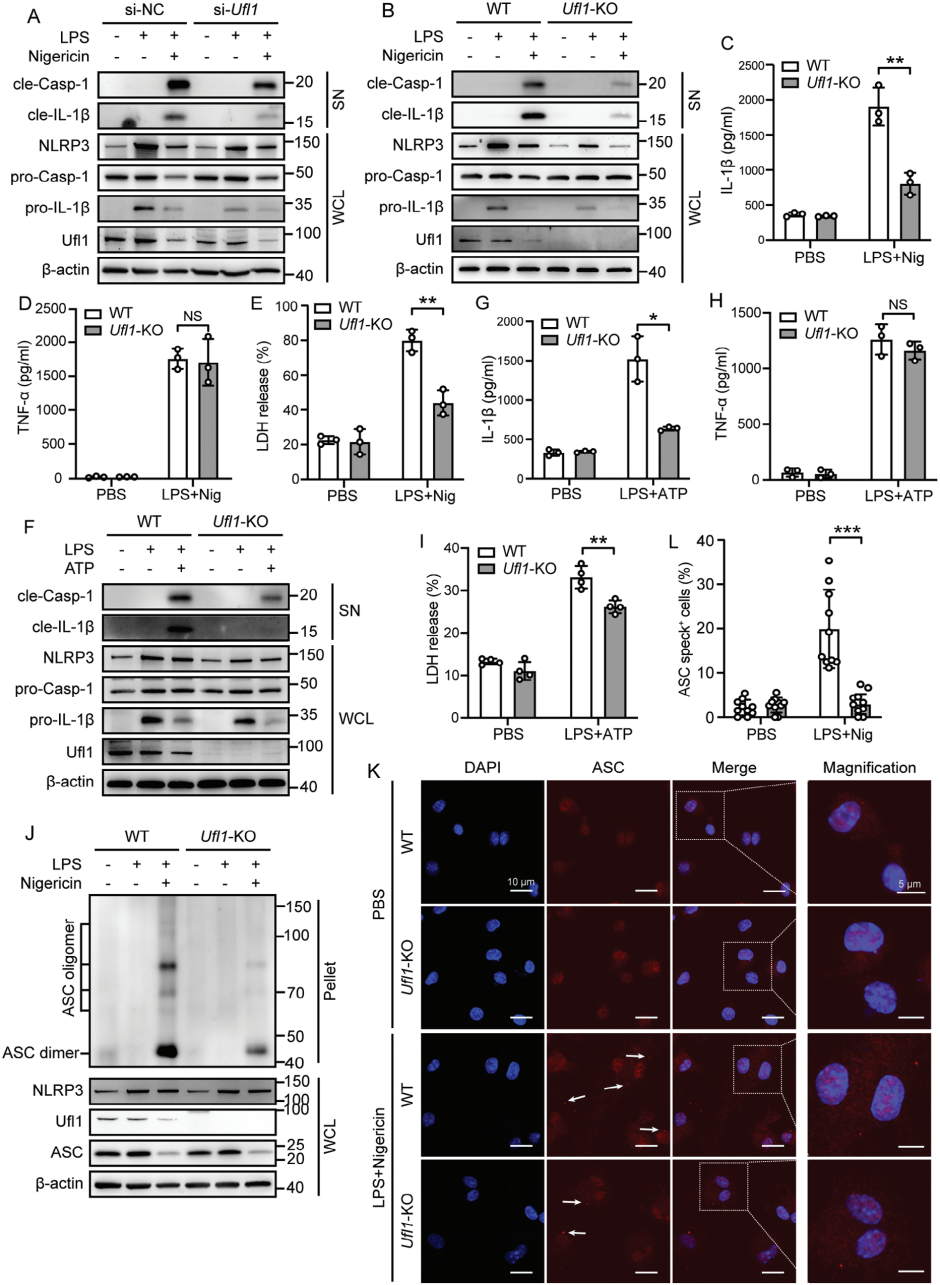
*Ufl1* deficiency inhibits the activation of NLRP3 inflammasome. A) Immunoblot analysis of proteins in supernatants (SN) and whole cell lysates (WCL) of LPS‐stimulated, LPS‐primed and nigericin‐activated iBMDMs transfected with si‐NC or si‐*Ufl1*. B) Immunoblot analysis of proteins in SN and WCL of LPS‐stimulated, LPS‐primed, and nigericin‐activated WT and *Ufl1*‐KO BMDMs. C–E) IL‐1β C), TNF‐α D) production and LDH release E) in SN of LPS‐primed and nigericin‐activated WT and *Ufl1*‐KO BMDMs. F) Immunoblot analysis of proteins in SN and WCL of LPS‐stimulated, LPS‐primed, and ATP‐activated (2 mm, 45 min) WT and *Ufl1*‐KO BMDMs. G–I) IL‐1β G), TNF‐α H) production, and LDH release I) in SN of LPS‐primed and ATP‐activated WT and *Ufl1*‐KO BMDMs. J) Immunoblot analysis of ASC oligomerization in pellets and WCL of LPS‐primed and nigericin‐activated WT and *Ufl1*‐KO BMDMs. K) Representative images of immunofluorescence analysis on LPS‐primed and nigericin‐activated WT and *Ufl1*‐KO BMDMs. Scale bar, 10 and 5 µm (magnification). L) Bar graph represents the percentage of cells exhibiting ASC speck (the percentage of ASC specks were counted in 5 different areas for each group). Data are representative of three independent experiments (A, B, F, J, and K). Data are shown as mean ± SD (C, D, E, G, H, I, and L). NS, no significance; *, *P* < 0.05; **, *P* < 0.01; ***, *P* < 0.001. *P* values were determined by unpaired two‐tailed Student's *t*‐test.

Next, we assessed whether *Ufl1* and *Ufm1* deficiency affected the activation of NLRP3 inflammasome. In LPS‐primed iBMDMs, after stimulated with NLRP3 inflammasome activators (nigericin), knockdown of *Ufl1* or *Ufm1* markedly inhibited caspase‐1 cleavage, IL‐1β maturation and secretion (**Figure** **2**A; and Figure S2A–C, Supporting Information). We further investigated the role of Ufl1 or Ufm1 in NLRP3 inflammasome activation by generating *Ufl1* or *Ufm1*‐conditional knockout mice (*Ufl1*‐cKO or *Ufm1*‐cKO). *Ufl1* or *Ufm1* was deleted in myeloid cells from mice which were generated by crossing mice with *lox*P‐flanked *Ufl1* alleles (*Ufl1* ^fl/fl^) with mice carrying the Cre recombinase inserted in the promoter of myeloid cell‐specific gene lysozyme M (*Lyz2*‐Cre). BMDMs from *Ufl1* ^fl/fl^ *Lyz2*‐Cre^−/−^ mice and *Ufl1* ^+/+^ *Lyz2*‐Cre^+/+^ mice exhibited no difference in NLRP3 inflammasome activation (Figure S2D, Supporting Information), indicating that *lox*P site insertion and lysozyme M‐driven Cre expression did not influence NLRP3 inflammasome activation. Hereafter, we chose *Ufl1* ^fl/fl^ *Lyz2*‐Cre^−/−^ mice as the WT control group in this study. We found that *Ufl1* deletion in BMDMs displayed reduced caspase‐1 cleavage and IL‐1β secretion after stimulation with LPS plus nigericin compared to WT BMDMs (Figure 2B,C), while *Ufl1* deficiency had no effects on tumor necrosis factor (TNF)‐α release (Figure 2D). In addition, NLRP3 inflammasome activation could trigger pyroptotic cell death, and we found cell death was also decreased in *Ufl1*‐knockout (*Ufl1*‐KO) BMDMs by detecting lactate dehydrogenase (LDH) release (Figure 2E). We also found similar results in *Ufl1‐*KO BMDMs stimulated with LPS plus ATP (Figure 2F–I). In addition, we analyzed apoptosis‐associated speck‐like protein containing a CARD (ASC) speck formation and ASC oligomerization, which are direct readouts of inflammasome activity. Fewer ASC specks and ASC oligomerization were observed in *Ufl1*‐KO BMDMs than those in WT cells after NLRP3 inflammasome activation (Figure 2J–L). Consistently, with the stimulation of LPS plus nigericin, *Ufm1*‐knockout (*Ufm1‐*KO) BMDMs showed reduced IL‐1β secretion, LDH release, caspase‐1 cleavage, and ASC oligomerization (Figure S2E–I, Supporting Information). Furthermore, we reconstructed NLRP3 inflammasome activation by coexpressed NLRP3, pro‐caspase‐1, ASC, and pro‐IL‐1β in HEK293T cells, and found that silence of *UFL1* decreased cleavage of pro‐IL‐1β and pro‐caspase‐1 (Figure S2J, Supporting Information), while overexpression of *UFL1* promoted inflammasome activation (Figure S2K, Supporting Information). In addition, *Ufl1* deletion in BMDMs did not affect the AIM2 inflammasome activation (Figure S2L–N, Supporting Information). These data indicate that *Ufl1* and *Ufm1* deficiency specifically result in reduced NLRP3 inflammasome activation.

### 2.3 Ufl1 Interacts with NLRP3 for Inflammasome Activation

We further explored the mechanism of Ufl1 in promoting NLRP3 inflammasome activation. Due to the role of Ufl1 and Ufl1‐mediated UFMylation in regulation of ER stress response in human granulosa‐like cells,^[^ ^38^ ^]^ cisplatin‐induced premature ovarian granulosa cells,^[^ ^39^ ^]^ tunicamycin‐induced bovine mammary epithelial cells,^[^ ^40^ ^]^ we first investigated whether the downregulation of NLRP3 inflammasome in macrophages was related to ER stress. We found the mRNA level of splicing of *XBP1* (*sXBP1*) increased in LPS‐stimulated *Ufl1*‐KO BMDMs compared to WT BMDMs, and other downstream genes of the ER stress response including *Ddit3* and *Atf4* did not show significant difference (Figure S3A, Supporting Information). However, the expression level of s*XBP1*, *Ddit3, and Atf4* in *Ufl1*‐KO BMDMs was much lower than that in WT BMDMs stimulated by the ER stress inducer, thapsigargin (Figure S3A, Supporting Information). In addition, *Ufl1* deletion also did not affect the level of downstream protein Grp75 of ER stress response pathway in LPS‐treated BMDMs (Figure S3B, Supporting Information). Therefore, we supposed that Ufl1 does affect the ER stress response upon LPS stimulation, but solely the IRE1 branch.

**Figure 3:**
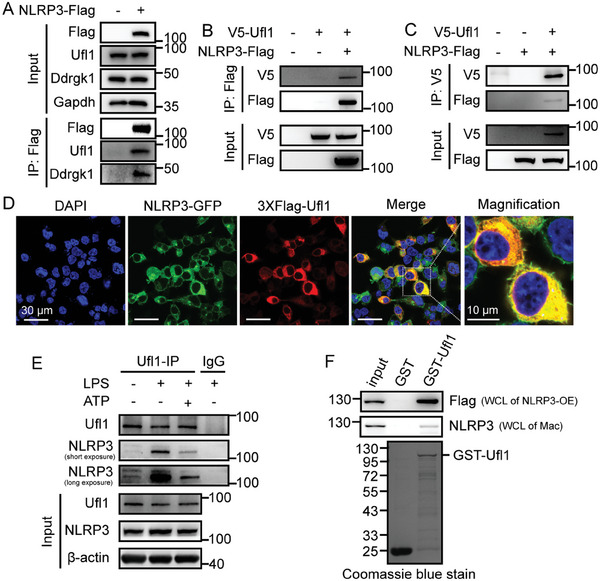
Ufl1 interacts with NLRP3 for inflammasome activation. A) Immunoblot analysis of lysates from HEK293T cells transfected with Flag vector or Flag‐tagged Nlrp3, and followed by immunoprecipitation (IP) with Flag beads. B,C) Immunoblot analysis of the association between NLRP3 and Ufl1 in HEK293T cells transfected with Flag‐tagged NLRP3 and V5‐tagged Ufl1, followed by IP with Flag beads B) and V5 antibody C). D) Representative images of immunofluorescence analysis of the association between C‐terminal GFP‐tagged NLRP3 (NLRP3‐GFP) (green) and 3 × Flag‐Ufl1 (red) in HEK293T cells. Scale bar, 30 and 10 µm (magnification). E) Immunoblot analysis of the endogenous association between NLRP3 and Ufl1 in LPS‐stimulated (1 µg mL^−1^, 3 h), LPS‐primed and ATP‐activated (2 mm, 30 min) THP1 cells, followed by IP with Ufl1 antibody. F) Immunoblotting of GST pull‐down products from cell lysates of HEK293T transfected with Flag‐tagged NLRP3 (WCL of NLRP3‐OE) with the anti‐Flag antibody, and cell lysates of LPS‐stimulated PM (WCL of Mac) with the anti‐NLRP3 antibody (Top). Coomassie blue staining of the GST proteins (Bottom). Data are representative of three independent experiments.

We speculated that Ufl1‐mediated protein UFMylation would regulate NLRP3 inflammasome activation. First, we examined whether Ufl1 interacted with NLRP3 inflammasome components. By immunoprecipitation assay, we found NLRP3 interacted with Ufl1 in HEK293T cells overexpressing Flag‐tagged NLRP3 (**Figure** **3**A). Also, we confirmed this interaction through coexpressing Flag‐tagged NLRP3 and V5‐tagged Ufl1 in HEK293T cells followed by coimmunoprecipitation (Figure 3B,C). Consistently, the confocal microscopy analysis demonstrated the colocalization between Ufl1 and NLRP3 in HEK293T cells overexpressing GFP‐tagged NLRP3 and 3 × Flag‐tagged Ufl1 (Figure 3D). Importantly, we detected the interaction between Ufl1 and NLRP3 in THP‐1‐derived macrophages in vivo by immunoprecipitation (Figure 3E). To further examine the interaction between Ufl1 and NLRP3 during NLRP3 inflammasome activation in vivo, we analyzed the association between NLRP3 and Ufl1 in THP‐1‐derived macrophages. We found the interaction between Ufl1 and NLRP3 occurred in resting state, increased in priming stage, while decreased in activation stage (Figure 3E). Furthermore, we observed the interaction between Ufl1 and NLRP3 in cell lysates from HEK293T transfected with Flag‐tagged NLRP3 and cell lysates from LPS‐stimulated PM through GST‐pull down assay (Figure 3F). Intriguingly, in our coimmunoprecipitation analysis, we also found that Ufl1 could slightly bind to IL‐1β but not ASC and caspase‐1 (Figure S3C, Supporting Information), which suggested that Ufl1 regulated NLRP3 inflammasome activation occurred in multiple processes including PTM of NLRP3 and assembly of inflammasome. Taken together, these data indicate that Ufl1 promotes NLRP3 inflammasome activation depending on the interaction with NLRP3.

### 2.4 NLRP3 is a New Substrate of UFMylation

**Figure 4:**
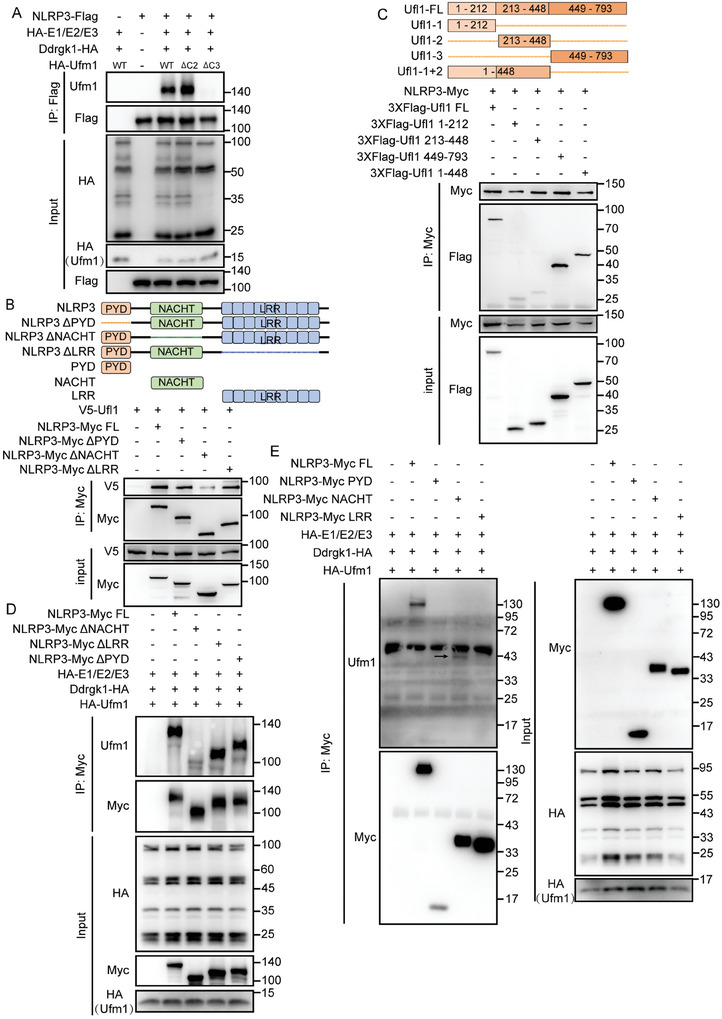
NLRP3 is a new substrate of UFMylation. A) IP analysis of NLRP3 UFMylation in HEK293T cells transfected with the indicated plasmids. B) Schematic diagram of NLRP3 and its truncation mutants (top), IP analysis of the interaction between NLRP3 or NLRP3 mutants and Ufl1 in HEK293T cells transfected with the indicated plasmids (bottom). C) Schematic diagram of Ufl1 and its truncation mutants (top); IP analysis of the association between NLRP3 and Ufl1 or Ufl1 mutants in HEK293T cells transfected with the indicated plasmids (bottom). D) IP analysis of UFMylation of NLRP3 mutants in HEK293T cells transfected with the indicated plasmids. E) IP analysis of UFMylation of NLRP3 mutants in HEK293T cells transfected with the indicated plasmids. The arrow indicates the specific band for UFMylation. Data are representative of three independent experiments. FL, full length.

Because Ufl1 is the only E3 ligase required for catalyzing protein UFMylation, we wondered whether Ufl1‐mediated UFMylation of NLRP3 was the potential mechanism in regulating NLRP3 inflammasome activation. First, we constructed the UFMylation system in HEK293T cells by overexpressing UFMylation components, including Uba5, Ufc1, Ufl1, Ufm1, and Ddrgk1 along with Flag‐tagged NLRP3, and found that WT Ufm1 and active form of Ufm1 with an exposed carboxy (C)‐terminal glycine residue (Ufm1‐∆C2) instead of inactive form of Ufm1 lacking the C‐terminal glycine residue (Ufm1‐∆C3) could conjugate to exogenously expressed NLRP3 (**Figure** **4**A; and Figure S4A, Supporting Information). Consistently, we observed Ufm1‐∆C3 almost abrogated the Ufl1‐mediated UFMylation of NLRP3 in HEK293T cells by overexpressing Myc‐tagged NLRP3 and 3 × Flag‐tagged Ufl1 (Figure S4B, Supporting Information). However, we failed to detect the UFMylation of other NLRP3 inflammasome components, including ASC, pro‐caspase‐1, and pro‐IL‐1β (Figure S4C, Supporting Information). These data demonstrate that NLRP3 is indeed a substrate of UFMylation mediated by Ufl1.

To explore which domain of NLRP3 could be UFMylated by Ufl1, we further constructed different NLRP3 truncations and domain‐deletion mutations. Deletion of NACHT domain (NLRP3‐ΔNACHT) in NLRP3 significantly decreased interaction between NLRP3 and Ufl1 (Figure 4B). We next constructed four truncations (1‐212 aa, 213–448 aa, 1–448 aa, 449–793 aa) of Ufl1 to examine which region of Ufl1 was required for binding to NLRP3 (Figure 4C). By coexpressing Myc‐tagged NLRP3 and the four Flag‐tagged truncations in HEK293T cells followed by immunoprecipitation, we found that individual truncation 1–212 aa and truncation 213–448 aa markedly reduced the interaction with NLRP3, indicating the structure and sequence of these two fragments are required for the interaction (Figure 4C). Furthermore, deletion of NACHT domain of NLRP3 (NLRP3‐ΔNACHT) significantly inhibited UFMylation of NLRP3 (Figure 4D). Consistently, we observed UFMylation in NACHT domain of NLRP3 by expressing three truncations containing pyrin domain (PYD), NACHT, leucine‐rich repeat (LRR) domain, respectively (Figure 4E), which suggested that NACHT domain might be vital for UFMylation of NLRP3. At the same time, the PYD domain and LRR domain, and the structure of NLRP3 protein are also crucial for NLRP3 UFMylation. Besides, we constructed three NACHT domain segment‐deletion truncated mutants, and the UFMylation assay showed that all three NACHT domain segment‐deletion decreased the UFMylation level of NLRP3 (Figure S4D, Supporting Information), highlighting that multiple sites of NACHT domain would be UFMylated by Ufl1. Therefore, we constructed mutants in which four lysine (K) residues were substituted with arginine (R) located in the NACHT domain. We found that all these four mutants (K320R, K375R, K426R, and K492R) significantly inhibited the UFMylation of NLRP3 (Figure S4E, Supporting Information). These data indicate that NACHT domain of NLRP3 is crucial for UFMylation and multiple lysine residues are UFMylated by Ufl1.

### 2.5 Ufl1 Prevents NLRP3 Degradation by Inhibiting Autophagy

**Figure 5:**
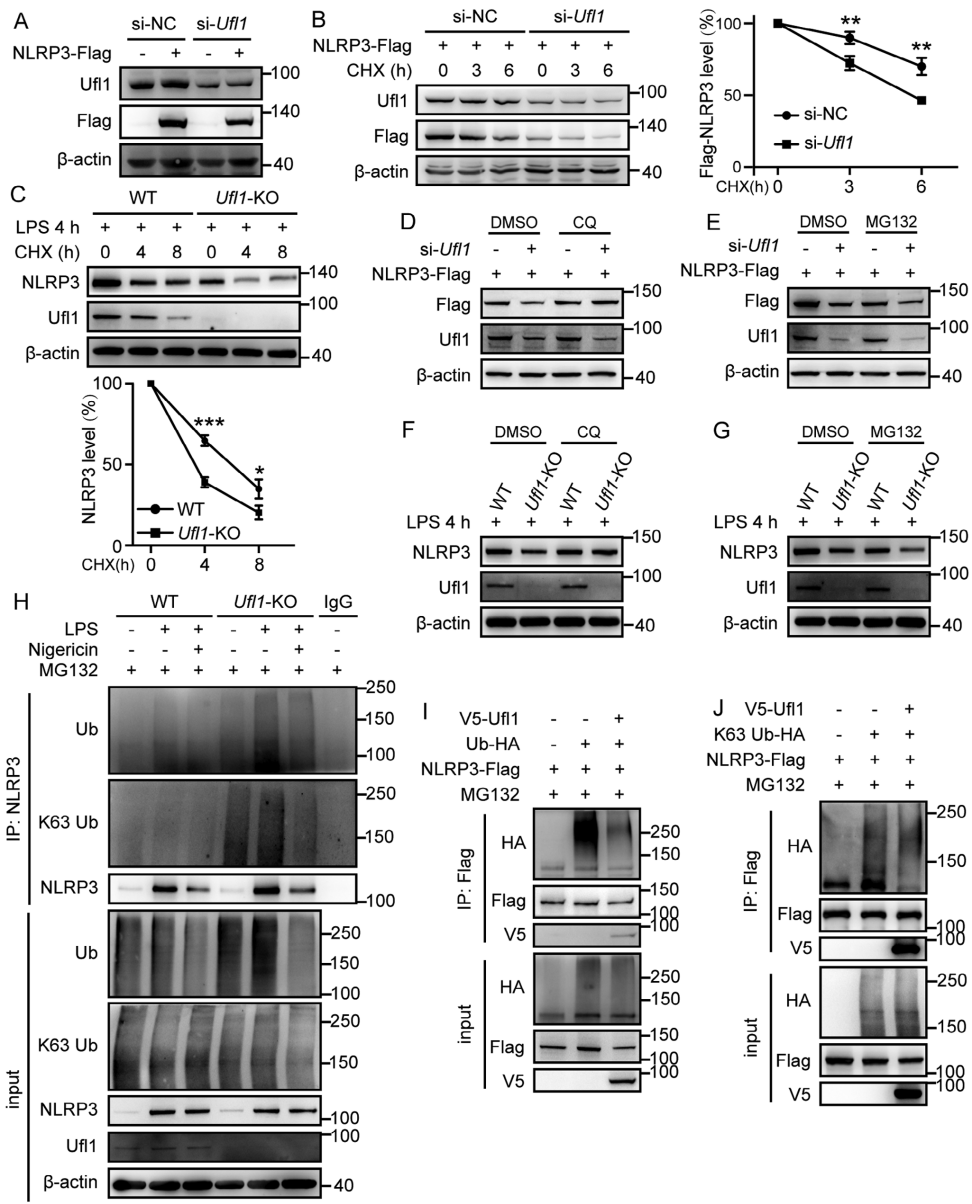
Ufl1‐mediated NLRP3 UFMylation suppresses its autophagy‐mediated degradation. A) Immunoblot analysis of HEK293T cells transfected with si‐NC or specific sequences targeting human UFL1 (si‐*UFL1*), and NLRP3‐Flag plasmid. B) Immunoblot analysis of lysates from HEK293T cells transfected with si‐NC or si‐*UFL1*, and NLRP3‐Flag plasmid, then treated with cycloheximide (CHX) (100 ng mL^−1^) for indicated time periods (left). NLRP3 expression was quantitated by measuring band intensities using ImageJ software (right). C) Immunoblot analysis of NLRP3 expression in WT and *Ufl1*‐KO BMDMs, following stimulation with LPS and then treated with CHX for indicated time periods (top). NLRP3 expression was quantitated by measuring band intensities using ImageJ software (bottom). D,E) Immunoblot analysis of NLRP3 expression in HEK293T cells transfected with si‐NC or si‐*UFL1*, and NLRP3‐Flag plasmid, following treatment of DMSO, CQ (50 µm) D) or MG132 (10 µm) E) for 8 h. F,G) Immunoblot analysis of NLRP3 expression in WT and *Ufl1*‐KO BMDMs treated with LPS for 4 h, following treatment of DMSO, CQ (50 µm) F) or MG132 (20 µm) G) for 6 h. H) Immunoblot analysis of ubiquitin and K63‐ubiquitin of lysates from WT and *Ufl1*‐KO PMs primed with LPS for 4 h, together with MG132 (10 µm) treatment for 3 h, and then stimulation with nigericin for 1 h, and followed by IP with NLRP3 antibody. I,J) Immunoblot analysis of lysates from HEK293T cells transfected with Ub‐HA I), or HA‐tagged K63‐linked ubiquitin J), NLRP3‐Flag, and V5‐Ufl1, following treatment with MG132 (10 µm) for 8 h and IP with Flag antibody. Data are representative of three independent experiments. Data are shown as mean ± SD (B, right panel; C, bottom panel). *, *P* < 0.05; **, *P* < 0.01; ***, *P* < 0.001. *P* values were determined by unpaired two‐tailed Student's *t*‐test.

The UFMylated lysine residues in NACHT domain were reported to be ubiquitinated, leading to the degradation of NLRP3 to inhibit its activation.^[^ ^41^, ^42^, ^43^ ^]^ Therefore, we speculated that Ufl1‐mediated UFMylation of NLRP3 would prevent the ubiquitination and subsequent degradation. We analyzed the stability of NLRP3 by silence or overexpression of *Ufl1*. *Ufl1* knockdown remarkably suppressed the NLRP3 protein levels (**Figure** **5**A). Correspondingly, the levels of NLRP3 protein increased by overexpression of *Ufl1* in a concentration‐dependent manner (Figure S5A, Supporting Information). More importantly, similar results were also observed in *Ufl1*‐KO BMDMs (Figure 2B,F). Interestingly, we found that the protein level of Ufl1 decreased faster than that of NLRP3 in the time‐course analysis of alterations in protein level (Figure S5B, Supporting Information). This observation indicated a potential negative feedback mechanism that prevents NLRP3 inflammasome from persistent activation by downregulating the UFMylation system. To further confirm Ufl1‐induced high levels of NLRP3 protein depending on preventing its degradation, we performed a cycloheximide (CHX) chase assay to detect half‐life of NLRP3 protein by inhibiting new protein synthesis. We found that Ufl1 knockdown accelerated degradation of NLRP3 protein after CHX treatment (Figure 5B), and *Ufl1* overexpression efficiently prolonged the half‐life of NLRP3 protein (Figure S5C, Supporting Information). In addition, the degradation rate of endogenous NLRP3 was faster in LPS‐primed *Ufl1*‐KO BMDMs than that in LPS‐primed WT BMDMs (Figure 5C). We further explored which protein degradation pathway was mainly blocked by NLRP3 UFMylation, and found that *Ufl1* deficiency‐mediated NLRP3 degradation was blocked by the autophagy inhibitor chloroquine (CQ) rather than the proteasome inhibitor MG132 (Figure 5D,E). Consistently, *Ufl1* deficiency‐mediated endogenous NLRP3 degradation in BMDMs was blocked by CQ rather than MG132 (Figure 5F,G). We further confirmed that *Ufl1* deficiency‐mediated NLRP3 degradation was rescued in *Beclin1*‐KO HEK293T cells (Figure S5D, Supporting Information). These together indicate that Ufl1‐suppressed NLRP3 degradation might mainly occur through the autophagy‐mediated pathway.

The above observations demonstrated Ufl1‐mediated UFMylation maintained the NLRP3 stability. Thus, we next assessed whether the ubiquitination of NLRP3 was affected by Ufl1 and found that *Ufl1* deficiency promotes NLRP3 ubiquitination in untreated, LPS‐primed, and nigericin‐activated PMs (Figure 5H). Especially, K63‐linked ubiquitination which is closely related with the autophagy‐mediated degradation^[^ ^44^ ^]^ was also increased in *Ufl1* deficient cells (Figure 5H). In addition, overexpression of *Ufl1* significantly decreased ubiquitination of NLRP3 in HEK293T cells expressing Flag‐tagged NLRP3, and HA‐tagged Ub or K63‐linked Ub (Figure 5I,J; and Figure S5E, Supporting Information). Consistently, silence of *Ufl1* facilitated ubiquitination of NLRP3 in HEK293T cells (Figure S5F, Supporting Information). And ectopic expression of total UFMylation system components efficiently attenuated the ubiquitination of NLRP3 (Figure S5G, Supporting Information). We next explored Ufl1‐mediated NLRP3 UFMylation could prevent autophagy of NLRP3. The autophagy marker LC3 displayed colocalization with NLRP3, and the number of NLRP3‐accosicated LC3 puncta increased in *Ufl1*‐knockdown HEK293T cells, which indicated autophagosomes containing NLRP3 accumulated while UFMylation was inhibited (Figure S5H, Supporting Information). Together, these data illustrate UFMylation of NLRP3 maintains its cellular levels by inhibiting autophagy‐mediated degradation.

### 2.6 Inhibition of UFMylation Ameliorates Inflammation In Vivo

**Figure 6:**
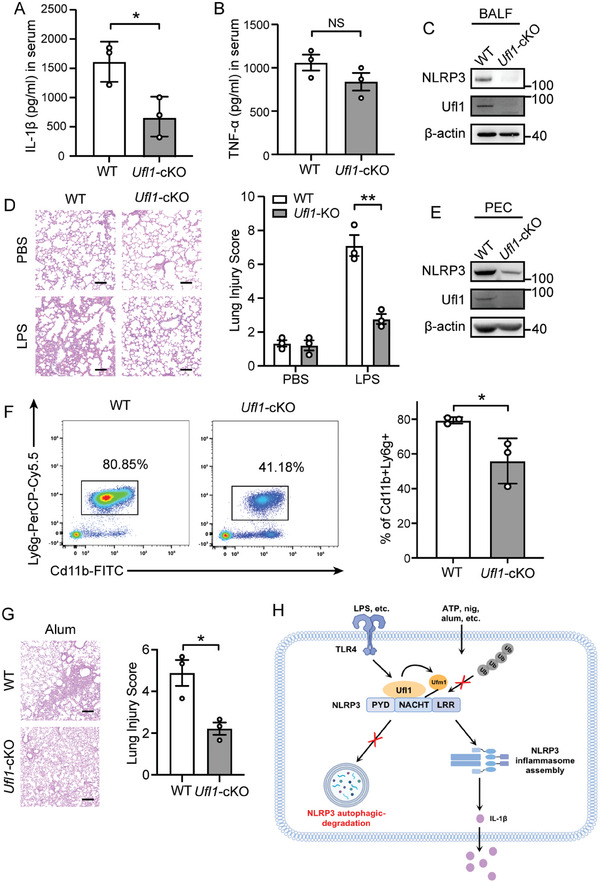
Ufl1 deficiency alleviates NLRP3 inflammasome activation in vivo. A–D) WT or *Ufl1‐*cKO mice (*n* = 3 per group) were intraperitoneally (i.p.) injected with lipopolysaccharide (LPS, 15 mg kg^−1^, 8 h). IL‐1β A) and TNF‐α B) release in serum was determined by ELISA. Immunoblot analysis of lysates from bronchoalveolar lavage fluids (BALFs) from WT or *Ufl1‐*cKO mice C). Representative images of H&E staining of lung tissues of WT and *Ufl1‐*cKO mice. Scale bar, 100 µm D). E–G) WT or *Ufl1*‐cKO mice (*n* = 3 per group) were i.p. injected with Alum (700 µg) for 12 h. Immunoblot analysis of lysates from peritoneal exudate cells (PECs) of WT or *Ufl1‐*cKO mice E). Flow cytometry analysis of CD11b^+^Ly6G^+^ neutrophils recruitment in blood from WT or *Ufl1‐*cKO mice F). Representative images of H&E staining of lung from WT and *Ufl1‐*cKO mice. Scale bar, 200 µm G). H) Graphic abstract showing the UFMylation of NLRP3 mediated by Ufl1, which maintains NLRP3 levels by suppressing autophagy‐mediated degradation, thereby sustaining the activation of NLRP3 inflammasome. Data are representative of three independent experiments D,G). Data are shown as mean ± SD A–G). NS: no significance; *, *P* < 0.05; **, *P* < 0.01. *P* values were determined by unpaired two‐tailed Student's *t*‐test.

We employed several NLRP3‐associated inflammatory disease models in mice to investigate the physiological effects of UFMylation in regulating NLRP3 inflammasome activation in vivo. Previous studies have reported that the IL‐1β production of intraperitoneal (i.p.) injection with LPS in mice was mainly from the activation of NLRP3 inflammasome,^[^ ^20^, ^45^ ^]^ so we compared the production of IL‐1β in WT and *Ufl1‐*cKO or *Ufm1‐*cKO mice after i.p. injection with LPS. In LPS‐induced endotoxic shock mice model, *Ufl1‐*cKO or *Ufm1‐*cKO mice displayed significantly reduced IL‐1β production in serum (**Figure** **6**A; and Figure S6A, Supporting Information), while there were no differences in the production of TNF‐α (Figure 6B; and Figure S6B, Supporting Information) compared to the littermate WT controls. Besides, NLRP3 protein levels decreased in the bronchoalveolar lavage fluids (BALFs) from the *Ufl1‐*cKO or *Ufm1‐*cKO mice induced by LPS (Figure 6C; and Figure S6C, Supporting Information). Lung tissues showed ameliorated infiltration of inflammatory cells in LPS‐treated knockout mice (Figure 6D; and Figure S6D, Supporting Information). Next, we constructed septic mice model by i.p. injection of *Escherichia coli* (*E. Coli*). In *Ufm1‐*cKO mice, we detected decreased spleen and liver colony‐forming units (CFU) (Figure S6E, Supporting Information), and ameliorated inflammation in lung tissues (Figure S6F, Supporting Information).

We also analyzed the role of Ufl1 in NLRP3 inflammasome‐driven peritonitis by i.p. injection of Alum. *Ufl1* deficiency in mice substantially downregulated the levels of NLRP3 protein in the peritoneal exudate cells (PECs) from mice with Alum challenge (Figure 6E). Additionally, we analyzed the infiltration of inflammatory cells in blood from Alum‐induced mice. *Ufl1*‐deficient mice exhibited reduced frequency of neutrophils (CD11b^+^Ly6G^+^) in blood (Figure 6F). Moreover, the ameliorated inflammation phenotypes were also observed in the lung tissues from Alum‐treated *Ufl1*‐cKO mice (Figure 6G). Collectively, these findings further demonstrated that *Ufl1* deficiency could suppress NLRP3 inflammasome activation in inflammatory‐related disease models in vivo. Taken together, these results demonstrate that UFMylation plays an essential role in sustaining NLRP3 inflammasome‐dependent inflammation in vivo.

In conclusion, our study identified the UFM1 E3 ligase Ufl1 can bind NLRP3 and UFMylate NLRP3. Meanwhile, Ufl1‐mediated UFMylation maintains the stability and cellular levels of NLRP3 by inhibiting autophagy‐mediated degradation, which is essential for sustaining the activation of NLRP3 inflammasome (Figure 6H). Our findings offer new insights for therapeutic targets for NLRP3 inflammasome‐related diseases.

## 3 Discussion

Given that NLRP3 inflammasome activation is correlated with the pathogenesis of various diseases, including neurological disorders,^[^ ^46^, ^47^, ^48^ ^]^ autoimmune diseases,^[^ ^49^, ^50^ ^]^ and metabolic diseases,^[^ ^51^, ^52^ ^]^ blocking the NLRP3 inflammasome activation pathway is widely considered an effective treatment for inflammatory diseases. However, the regulatory mechanisms of NLRP3 inflammasome activation are still not completely understood. Increasing evidence has demonstrated that PTMs are critical for regulation of NLRP3 inflammasome activation, while whether UFMylation, a newly identified PTM, mediates the activation of NLRP3 is currently unknown. In this study, we illustrated UFMylation functions as an important PTM of NLRP3 for its activation. We further demonstrate that Ufl1 binds to and catalyzes UFMylation of NLRP3 to avoid autophagic degradation of NLRP3.

Given the importance of NLRP3 inflammasome in multiple diseases, the priming and activation of inflammasome must be tightly controlled. During the priming of NLRP3 inflammasome, it can be regulated at least by two aspects, including modulating the expression of the inflammasome components, such as NLRP3, pro‐IL‐1β, and caspase1, and stabilizing NLRP3 proteins in a certain level with inactive state through inducing various PTMs.^[^ ^5^ ^]^ In our study, we found that the deficiency of *Ufl1* or *Ufm1* could suppress the expression of *Il1b* and *Nlrp3*. Meanwhile, we also found that the protein level of NLRP3 was sustained by Ufl1‐mediated UFMylation. Therefore, the role of Ufl1 and its mediated UFMyaltion are important for the priming of NLRP3 inflammasome.

UFMylation is one of the ubiquitin‐like PTMs, which is involved in various biological processes,^[^ ^25^ ^]^ including ER homeostasis,^[^ ^28^, ^53^ ^]^ DNA damage repair,^[^ ^54^ ^]^ and hematopoietic stem cell function.^[^ ^55^ ^]^ In addition, dysfunction and mutations of UFMylation system have been reported to be closely associated with many diseases.^[^ ^24^, ^56^, ^57^, ^58^, ^59^ ^]^ More recently, UFL1 and UFMylation have been found to regulate immune responses. For example, UFMylation inhibits the proinflammatory capacity of interferon‐γ (IFN‐γ)‐activated macrophages.^[^ ^35^ ^]^ UFL1 promotes antiviral innate immune responses by inducing UFMylation of RIG‐I^[^ ^36^ ^]^ and maintaining STING stability^[^ ^34^ ^]^ thereby facilitating type I IFN production. UFMylation of PD‐L1 promotes the antitumor immunity by mediating degradation of PD‐L1.^[^ ^33^ ^]^ In addition, UFL1 in CD8^+^ T cells promotes UFMylation of PD‐1 to block its degradation. Depletion of UFL1 in T cells effectively enhances CD8^+^ T cells activation and antitumor immunity.^[^ ^60^ ^]^ Our study further illustrated the function and regulatory mechanism of UFMylation in inflammation regulation, lack of *Ufl1* and *Ufm1* significantly inhibited the activation of NLRP3 inflammasome. These findings emphasize UFMylation system would be a potential therapeutic target for the treatment of inflammatory diseases. While different from ubiquitination, the study of UFMylation still lacks powerful tools, like UFM1‐detecting probes to interrogate the enzymatic processes and effective structural domain of the E3 ligase Ufl1.^[^ ^61^ ^]^ Further investigations are urgently required to deeply and comprehensively explore the molecular mechanisms in UFMylation dynamics, which will undoubtedly promote the clinical translation by targeting UFMylation system for inflammatory disease treatment.^[^ ^61^, ^62^, ^63^ ^]^

The crosstalk among different types of PTMs on NLRP3 has been reported to be involved in NLRP3 inflammasome activation.^[^ ^64^, ^65^, ^66^ ^]^ In this study, we identified that the core component of NLRP3 inflammasome, NLRP3, could be modified by UFMylation, and we found NLRP3 UFMylation prevents the ubiquitination of NLRP3. This molecular mechanism was consistent with previous findings in UFMylation of tumor suppressor p53, which maintained its stability by antagonizing the ubiquitination.^[^ ^32^ ^]^ In regulation of NLRP3 activity, TRIM28 binds to NLRP3, inducing SUMOylation of NLRP3, thereby inhibits NLRP3 ubiquitination and stabilizes NLRP3.^[^ ^67^ ^]^ HERCs (the predominant E3 ISGylation ligases) promotes NLRP3 ISGylation and inhibits K48‐linked ubiquitination to facilitate inflammasome activation.^[^ ^68^ ^]^ The abnormal crosstalk in PTMs is closely related to pathogenesis of various diseases.^[^ ^69^ ^]^ Therefore, precise regulation of NLRP3 inflammasome activation by the crosstalk of UFMylation and ubiquitination may be one of the potential ways for the control of inflammation. Besides, we demonstrated that the NACHT domain is important for the NLRP3 UFMylation, and mutations in multiple lysine residues in NACHT domain could inhibit NLRP3 UFMylation. Importantly, the ATPase activity sites in the NACHT domain have been reported as a therapeutic target for NLRP3‐related diseases,^[^ ^70^ ^]^ which further supported the potential function of NLRP3 UFMylation for the inflammatory diseases’ treatment. However, it remains to be explored whether the UFMylation sites of NLRP3 take place on the same residue with ubiquitination and whether the sites have a sequential modification.

Recently, an increasing number of studies have shown that autophagy plays an important role in NLRP3 inflammasome activation in the development of various inflammatory diseases.^[^ ^71^, ^72^, ^73^, ^74^ ^]^ Autophagy suppresses NLRP3 inflammasome activation by removing inflammasome activators, such as ROS, damaged mitochondria, cytokines, and inflammasome components.^[^ ^20^, ^71^, ^75^, ^76^ ^]^ Furthermore, some types of PTMs lead NLRP3 degradation through triggering autophagy pathways. For instance, USP5 promotes NLRP3 deubiquitination through autophagy‐lysosomal degradation pathway.^[^ ^77^ ^]^ NLRP3 palmitoylation significantly promotes NLRP3 degradation dependent on lysosome‐autophagic manner through enhancing the interaction between NLRP3 and autophagy‐chaperone, HSC70.^[^ ^20^ ^]^ In addition, PTPN22‐mediated phosphorylation of NLRP3 can promote the degradation of NLRP3 in autophagy‐dependent pathway.^[^ ^78^ ^]^ In some pathological conditions, autophagy has been reported as one of major mechanisms for limiting excessive activation of NLRP3. IRGM, a risk factor of Crohn's disease, directly interacts with NACHT domain of NLRP3, promotes the selective degradation of NLRP3 in p62‐dependent way, therefore inhibit the intestinal inflammation.^[^ ^79^ ^]^ Here, we clarified that Ufl1‐mediated NLRP3 UFMylation prevents NLRP3 levels and inflammasome activation by suppressing autophagic degradation of NLRP3. Together with the previous studies, we highlight the importance of autophagy involved in NLRP3 inflammasome activation.

In summary, we demonstrated that UFMylation, a new PTM of NLRP3, can maintain NLRP3 stability through inhibiting autophagy‐mediated degradation of NLRP3, thereby sustaining the NLRP3 inflammasome activation. Our study highlights the importance of Ufl1 and it‐mediated UFMylation in regulation of NLRP3 inflammasome activation, providing new insights into potential therapeutic target for NLRP3 inflammasome‐triggered diseases.

## 4 Experimental Section

### Mice

*Ufl1*‐flox mice were described previously.^[^ ^80^ ^]^ *Ufm1*‐flox mice were purchased from Cyngen (S‐CKO‐17583), in which the *Ufm1* exon3 and exon4 were flanked by *lox*P sites. *Lyz2‐*Cre mice were purchased from the Jackson Laboratory. *Ufm1*‐flox or *Ufl1*‐flox mice were crossed with *Lyz2‐*Cre mice to generate *Ufm1‐*cKO or *Ufl1‐*cKO mice with *Ufm1* or *Ufl1* deficiency in myeloid cells, respectively. The background strain of all mice used is C57BL/6J. This study was approved by the Institutional Animal Care and Use Committee at Tongji University (approval number: TJ‐HB‐IAC‐2023‐11). All mice were housed in a specific‐pathogen free (SPF) environment with the standard conditions of temperature maintained at 20–26 °C, relative humidity at 40%–70% in the Tongji University animal facilities, and all mice were 8–12 weeks of age when used.

### Reagents

Antibodies against DDDDK/Flag (AE005), HA (AE008), Myc (AE070), p‐IκBα‐S32 (AP0707), p‐Erk (AP0974), ASC (A22046) were purchased from ABclonal Technology. Antibody against Ufl1 (HPA030559) and glutathione (1 294 820) were purchased from Sigma‐Aldrich. Antibody against Ufm1 (ab109305) was purchased from Abcam. Antibodies against NLRP3 (AG‐20B‐0014‐C100), Caspase‐1 (AG‐20B‐0042) were purchased from AdipoGen Life Science. Antibody against IL‐1β (AF‐401‐NA) was purchased from R&D Systems. Antibody against β‐actin (GNI4110‐BA) was purchased from GNI Group. Antibodies against p‐p65 (3033S), p65 (8242S), IκBα (4812S), Erk (4695S), p‐JNK (4668S), JNK (4252S), Ubiquitin (P4D1) (3936S), K63‐linkage Specific Polyubiquitin (D7A11) (5621S), and V5 (D3H8Q) (13202S) were purchased from Cell Signaling Technology. Antibody against Beclin 1 (JE59‐31) was purchased from HuaBio. Anti‐c‐Myc Affinity Beads (SA065001), Protein A/G beads (SA032005), and Glutathione Sepharose beads (SA008005) were purchased from Smart‐Lifesciences. Antibody against ASC (SC‐514414) for immunofluorescence was purchased from Santa Cruz Biotechnology. Antibody against LC3 (NB100‐2220) for immunofluorescence was from Novus Biologicals. LPS (tlrl‐rslps), ATP (tlrl‐atpl), nigericin (tlrl‐nig), poly(dA:dT) (tlrl‐patn), and Alum (tlrl‐aloh) were purchased from InvivoGen. CHX (HY‐12320) was purchased from MCE. MG132 (T2154) and thapsigargin (TQ0302) were purchased from TargetMol. Chloroquine (CM02020) was purchased from Proteintech.

### Cells and Culture Method

Bone marrow (BMs) cells were isolated from WT, *Ufm1‐*cKO, and *Ufl1‐*cKO mice, then BMs were differentiated into bone marrow‐derived macrophages (BMDMs), showed as WT & KO‐BMDMs in this study, cultured in DMEM medium supplemented with 10% fetal bovine serum (FBS), 1% Penicillin‐Streptomycin (PS), and 25 ng mL^−1^ recombinant mouse macrophage colony‐stimulating factor (M‐CSF). On day 5, BMDMs were used to perform related experiments in this study.

To obtain mouse primary peritoneal macrophages (PMs), WT and *Ufl1‐*cKO mice were injected i.p. with 3% Brewer's thiogly‐collate broth. After 3 days, peritoneal exudate cells were harvested and seeded, cultured in DMEM medium supplemented with 10% FBS and 1% PS.

The human embryonic kidney (HEK) 293T cells and the immortalized bone marrow‐derived macrophages (iBMDMs) were cultured in DMEM medium supplemented with 10% FBS and 1% PS.

All cells were maintained in a 5% CO_2_ environment at 37 °C as described in the method details section below.

### NLRP3 and AIM2 Inflammasome Activation

iBMDMs and BMDMs were stimulated with LPS (500 ng mL^−1^, 4 h) and ATP (2 mm, 45 min), or LPS (200 ng mL^−1^, 4 h) and nigericin (10 µm, 1 h) to induce the NLRP3 inflammasome activation. BMDMs were stimulated with LPS (200 ng mL^−1^, 4 h) and poly(dA:dT) (2 µg mL^−1^, 6 h) to induce the AIM2 inflammasome activation.

### Plasmid Construction and Transfection

HA‐tagged Uba5, Ufc1, Ufl1, Ddrgk1 plasmids were kindly provided by Dr. Yusheng Cong (Hangzhou Normal University, Hangzhou, Zhejiang, China). Myc‐tagged NLRP3 and its truncated fragments plasmids were kindly provided by Dr. Wei Zhao (Shandong University, Jinan, Shandong, China). Flag‐tagged NLRP3, pro‐Caspase‐1, pro‐IL‐1β, ASC plasmids, 3 × Flag‐tagged Ufl1, and its truncated fragments plasmids, and V5‐tagged Ufl1 plasmid were constructed by polymerase chain reaction (PCR)‐based amplification, and then subcloned into the pcDNA3.1 eukaryotic expression vector. All constructs were confirmed by DNA sequencing. Plasmids were transfected into HEK293T cells with jetPRIME transfection reagent (Polyplus).

### ELISA and LDH Assay

Cell culture supernatants and sera were used for ELISA to detect IL‐1β (Invitrogen) and TNF‐α (Proteintech) protein levels. Cytotoxicity was measured by a lactate dehydrogenase (LDH) assay kit from Promega Corporation according to the manufacture instructions.

### Quantitative Real‐Time RT‐PCR (qPCR)

Total RNA was prepared using a total RNA_fast200_ kit (fastagen 220 010) for genes expression analysis. Complementary DNA (cDNA) was synthesized using Evo M‐MLV RT Premix for qPCR (Accurate Biology, AG11706). Real‐time qPCR was performed using SYBR Green Premix Pro Taq HS qPCR Kit (Accurate Biology, AG11701). Relative mRNA expression was assessed using the 2^−ΔΔCt^ method and normalized to the housekeeping gene *Actb*. Primer sequences are provided in **Table** **1**.

**Table 1:** Sequences of PCR primers used in this study.

Name	Prime	Sequence
m*Atf4*	Forward	5′‐ATGAGCCCAGAGTCCTACCT‐3′
	Reverse	5′‐CATAAGGTTTGGGCCGAGGA‐3′
m*Ddit3*	Forward	5′‐CCCAGGAGGAAGAGGAGGAA‐3′
	Reverse	5′‐CTTCATGCGTTGCTTCCCAG‐3′
m*NLRP3*	Forward	5′‐CTGGTGACTTTGTATATGCGTG‐3′
	Reverse	5′‐GCTTAGGTCCACACAGAAAGT‐3′
m*Il‐1β*	Forward	5′‐GCAACTGTTCCTGAACTCAACT‐3′
	Reverse	5′‐ATCTTTTGGGGTCCGTCAACT‐3′
m *sXBP1*	Forward	5′‐GCTGAGTCCGCAGCAGGT‐3′
	Reverse	5′‐CAGGGTCCAACTTGTCCAGAAT‐3′
m *uXBP1*	Forward	5′‐CAGACTACGTGCACCTCTGC‐3′
	Reverse	5′‐CAGGGTCCAACTTGTCCAGAAT–3′
m*Actb*	Forward	5′‐GGCTGTATTCCCCTCCATCG‐3′
	Reverse	5′‐CCAGTTGGTAACAATGCCATGT‐3′

### Western Blot

Proteins from cells and tissues were extracted by using cell lysis buffer (Cell Signaling Technology), and then were diluted in 5 × loading buffer (Sangon Biotech) to boil at 100 °C. Protein concentration of tissue lysates was measured with bicinchoninic acid (BCA) assay. The supernatants were chloroform‐methanol precipitated and 1 × loading buffer was added before boiling. Femto‐sig ECL Western Blotting Substrate (Tanon) and Tanon 4600 system were used to visualize the antibody‐antigen complex.

### RNA Interference

iBMDMs or HEK293T cells were plated in 24‐well plate for 12 h before transfection. Cells were transfected with specific siRNAs targeting *Ufl1* and *Ufm1* by INTERFERin (Polyplus‐transfection) at 30%–50% confluency and the medium was replaced 4 h after transfection. Cells were stimulated with LPS after 24 h. siRNAs were synthesized as follows: human *UFL1*: 5′‐GUUCCAACAUCGACAAGCA‐3′ (30), murine *Ufl1*: 5′‐GCAGCCAUUACAAGUGAUATT‐3′, murine *Ufm1*: 5′‐GCAGCUACAAGUGCGAUUATT‐3′, negative control: 5′‐UUCUCCGAACGUGUCACGUTT‐3′.

### Immunofluorescent Confocal Analysis

For ASC speck staining, BMDMs were plated on cover glasses in 6‐well plate. After stimulation, cells were washed with pre‐cold PBS and fixed with 4% paraformaldehyde for 15 min at room temperature (RT). After a wash with PBS, cells were permeabilized with 0.5% Triton X‐100 for 10 min at RT. After another wash with PBS, 5% BSA was used for blocking at RT for 30 min. Cells were then incubated with anti‐ASC antibody (1:100, Santa Cruz) overnight at 4 °C. The next day, cells were washed with PBS and incubated away from light with Alexa Fluor 555‐labeled Donkey Anti‐Mouse IgG (1:500, Beyotime) for 1 h at RT. At last, nuclei were stained with DAPI (Beyotime). Nikon laser scanning confocal microscope system was used to capture ASC specks.

To detect the colocalization of NLRP3 with Ufl1 or LC3, HEK293T cells transfected with indicated plasmids were plated on cover glassed in 6‐well plate. Cells were incubated with primary antibodies and then stained with Alexa Fluor 555‐labeled Donkey Anti‐Mouse IgG (1:500, Beyotime) or Alexa Fluor 555‐labeled Donkey Anti‐Rabbit IgG (1:500, Beyotime) for 1 h at RT. Colocalization was visualized with Nikon laser scanning confocal microscope system and analyzed using ImageJ software.

### ASC Oligomerization Assay

To detect the oligomerization of ASC, BMDMs were lysed in cell lysis buffer (50 mm Tris, pH 7.5, 150 mm NaCl, 0.5% Triton‐X100, and protease inhibitor cocktail) for 30 min at 4 °C, then centrifuged at 6000 g at 4 °C for 15 min. Supernatants were discarded and the pellets were resuspended in 200 µL PBS containing 2 mm of disuccinimidyl suberate (dissolved in DMSO). The pellets were incubated for 30 min at RT and centrifuged at 6000 g at 4 °C for 15 min. Supernatants were discarded and the pellets were dissolved in the loading buffer. Samples were heated for 15 min at 100 °C.

### Protein Purification

For GST tagged protein purification, pGEX‐4T‐1 plasmid containing Ufl1 was transformed into *E. coli* (BL21). *E. coli* were grown to OD600 of 0.6–0.8 and were induced with 0.2 mm IPTG at 18 °C overnight. Bacteria were harvested and lysed in lysis buffer (300 mm NaCl, 25 mm Tris‐HCl pH7.4, 2 mm DTT, 1 mm PMSF). The supernatant was harvest after centrifugation at 12 000 rpm for 30 min at 4 °C. GST tagged proteins were purified on Glutathione Sepharose beads and eluted with 10 mm glutathione in lysis buffer. Purified protein was detected by coomassie blue staining.

### GST Pull Down Assay

GST and GST‐tagged Ufl1 were expressed in *E. coli* (BL21) and purified by Glutathione Sepharose beads. HEK293T cells transfected with Flag‐tagged NLRP3, LPS‐stimulated macrophages were harvested and were lysed in lysis buffer (150 mm NaCl, 50 mm Tris‐HCl pH7.4, 1% Trition X‐100, 1 mm EDTA, and protease inhibitor). After washed three times, the Glutathione Sepharose beads were incubated with cell lysates with rotation at 4 °C overnight. The beads were washed three times with lysis buffer plus protease inhibitor cocktail. Samples were boiled in SDS‐loading buffer at 100 °C for 5 min and analyzed by Western blotting.

### In Vivo Mice Inflammatory Model Construction

To analyze LPS‐induced endotoxemia, WT, *Ufm1‐*cKO, and *Ufl1‐*cKO mice at 8–12 weeks were intraperitoneally (i.p.) injected with LPS (15 mg kg^−1^) for 8 h. Sera were collected to test proinflammatory cytokines, lung tissues were harvested for hematoxylin & eosin (H&E) staining, and bronchoalveolar lavage fluids (BALF) were collected for immunoblot assay.

To analyze Alum‐induced peritonitis, WT and *Ufl1‐*cKO mice at 8–12 weeks were i.p. injected with 700 µg Alum in 100 µL PBS for 12 h. Lung tissues were collected for H&E, and blood was collected for flow cytometry analysis.

To analyze *E. coli‐*induced peritonitis, WT and *Ufm1‐*cKO mice at 8–12 weeks were i.p. injected with *E. coli* (1 × 10^9^ kg^−1^ body weight) for 24 h. Lung tissues were collected for H&E staining, and spleens were collected and lysed for measurement of colony‐forming units (CFU).

### Flow Cytometry Analysis

Cells in blood from mice were collected and stained with antibodies against mouse CD11b and Ly6G in PBS at 4 °C away from light for 30 min. After washing twice with PBS, the cells were suspended in 300 µL PBS and detected using Cytek Aurora (Cytek Biosciences). Flow cytometry data were analyzed by SpectroFlo software (Cytek Biosciences).

### Immunoprecipitation and Ubiquitination Assay

For IP, HEK293T cells were transfected with specific plasmids by jetPRIME transfection reagent (Polyplus) for 36–48 h, THP‐1‐derived macrophages were stimulated with LPS (1 µg mL^−1^, 3 h) and ATP (2 mm, 30 min). And then, cells were harvested and lysed in 0.3% NP‐40 lysis buffer. The cell lysates were immunoprecipitated with anti‐Flag, anti‐c‐Myc beads for HEK293T cells for 3 h, or protein G Plus‐Agarose IP reagent together with anti‐NLRP3, anti‐Ufl1 for THP‐1‐derived macrophages overnight at 4 °C.

For ubiquitination assay, transfected HEK293T cells were lysed in hot lysis buffer (0.3% NP‐40 buffer, 5 mm EDTA, 10% SDS) and immediately boiled for 15 min. Cell lysates were then diluted ten times with NP‐40 buffer and subjected to immunoprecipitation using the indicated antibodies overnight at 4 °C and then incubated with protein A/G beads for 3 h at 4 °C and finally blotted with specific antibodies.

### UFMylation Assay

For UFMylation assay, transfected HEK293T cells were lysed in buffer (150 mm Tris‐HCl (pH 8.0), 5% SDS, and 30% glycerol) and immediately boiled for 10 min. Cell lysates were diluted 20 times with buffer A (50 mm Tris‐HCl (pH 8.0), 150 mm NaCl, 0.5% NP‐40, and 2 mm *N*‐ethylmaleimide) for anti‐c‐Myc IP experiments; or buffer A (6 m guanidine‐HCl, 0.1 m Na_2_HPO_4_/NaH_2_PO_4_, and 10 mm imidazole, pH 8.0) for anti‐HA IP experiments, and protease inhibitor cocktails. After incubation with anti‐c‐Myc beads or anti‐HA beads for 3 h at 4 °C, the cell lysates were finally blotted with specific antibodies.^[^ ^32^ ^]^

### Statistical Analysis

Data are presented as mean ± standard deviation for all results from at least three repeated experiments. Statistical analysis was performed using two‐tailed unpaired Student's *t*‐test. Statistical significance was considered at *P* < 0.05 (*, *P* < 0.05; **, *P* < 0.01; ***, *P* < 0.001; ****, *P* < 0.0001). The above analyses were carried out with GraphPad Prism 9 (GraphPad Software Inc., La Jolla, CA). The detailed statistical analysis and sample size applied to each experiment is presented in the corresponding figure legends.

## Conflict of Interest

The authors declare no conflict of interest.

## Author Contributions

J.J.J., F.Y., and K.W. contributed equally to this work. K.C. and D.Q.D. planned the project. J.J.J., F.Y., and K.W. conducted the experiments. M.T.C. assisted with the experiments. N.K. performed the construction of inflammatory mice models. S.X.W. supported in ELISA experiments. X.Y.Q. supported in WB experiments. F.Y.K. supported in qPCR experiments. D.Y.Z. and J.L.J. supported in animal experiments. L.X.T. and J.X.G. helped in experiments design. J.J.J., F.Y., and K.C. wrote the manuscript. J.J.J., F.Y., and Y.‐S.C. analyzed the data. D.Q.D. discussed the results. K.C. supervised the study.

## Supporting information



Supporting Information

Supporting Table 1

## Data Availability

Data sharing not applicable‐no new data generated.
