# Supplementary material for: UFMylation of NLRP3 Prevents Its Autophagic Degradation and Facilitates Inflammasome Activation
Source: Adv Sci (Weinh). 2025 Feb 22;12(15):2406786. doi: 10.1002/advs.202406786 (PMC12005806; doi:10.1002/advs.202406786)
Supplement: Supplementary file 1 — Supporting Information [file ADVS-12-2406786-s001.docx]

**Supporting Information for**

**UFMylation of NLRP3 Prevents Its Autophagic Degradation and Facilitates Inflammasome Activation**

**Authors:**

Jiongjie Jing^1#^, Fan Yang^1#^, Ke Wang^2#^, Mintian Cui^1^, Ni Kong^1^, Shixi Wang^1^, Xiaoyue Qiao^1^, Fanyu Kong^3^, Dongyang Zhao^3^, Jinlu Ji^3^, Lunxian Tang^3^, Jiaxin Gao^4^, Yu-Sheng Cong^5^, Deqiang Ding^2, 6*^, Kun Chen^1, 6*^

**Affiliations:**

1. State Key Laboratory of Cardiovascular Diseases and Medical Innovation Center, Shanghai East Hospital, School of Life Sciences and Technology, Tongji University, Shanghai 200127, China.

2. Shanghai Key Laboratory of Maternal Fetal Medicine, Clinical and Translational Research Center of Shanghai First Maternity and Infant Hospital, School of Life Sciences and Technology, Tongji University, Shanghai 200092, China.

3. Department of Internal Emergency Medicine, Shanghai East Hospital, School of Medicine, Tongji University, Shanghai 200120, China.

4. State Key Laboratory of Mycology, Institute of Microbiology, Chinese Academy of Sciences, Beijing 100101, China.

5. Key Laboratory of Aging and Cancer Biology of Zhejiang Province, Institute of Aging Research, School of Medicine, Hangzhou Normal University, Hangzhou 311121, China.

6. Shanghai Key Laboratory of Signaling and Disease Research, Frontier Science Center for Stem Cell Research, School of Life Sciences and Technology, Tongji University, Shanghai 200092, China.

#J.J. Jing, F. Yang, and K. Wang contributed equally to this paper; *D.Q. Ding and K. Chen contributed equally to this paper. Correspondence to: K. Chen: chenk@tongji.edu.cn (K. C.), D.Q. Ding; dingdeqiang@tongji.edu.cn (D. D.).

**Supplemental Figures.**


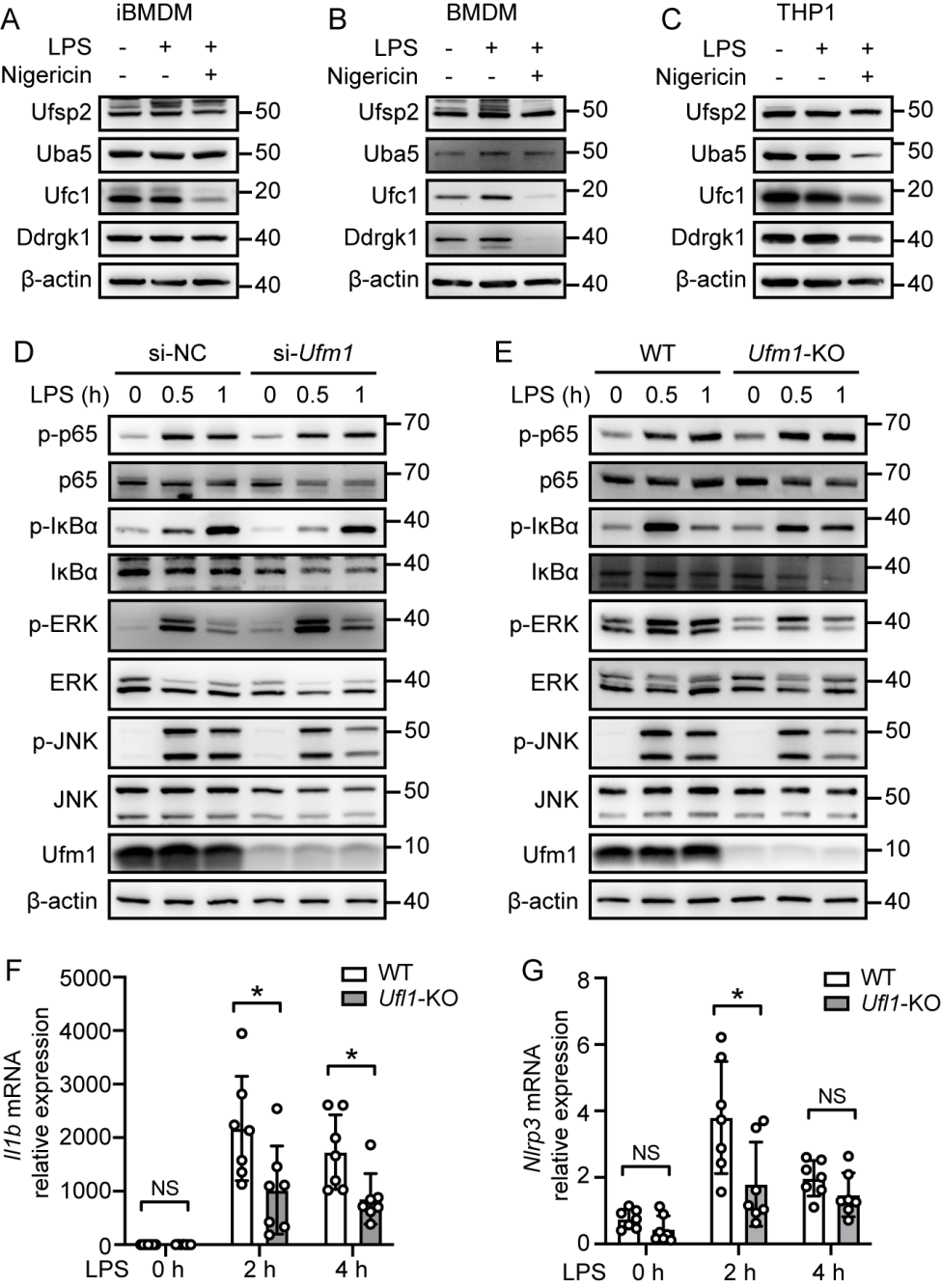


**Figure S1. *Ufm1* deficiency suppresses MAPK signaling pathway after LPS treatment. (A-C)** Immunoblot analysis of core components of UFMylation in the whole cell lysis of LPS-stimulated, LPS-primed and nigericin-activated iBMDMs (A), BMDMs (B) and THP1 (C). **(D)** Immunoblot analysis of p-p65, p65, p-IκBα, IκBα, p-ERK, ERK, p-JNK, and JNK in the whole cell lysis of LPS-stimulated iBMDMs transfected with si-NC or specific sequences targeting Ufm1 (si-*Ufm1*). **(E)** Immunoblot analysis of p-p65, p65, p-IκBα, IκBα, p-ERK, ERK, p-JNK, and JNK in the whole cell lysis of LPS-stimulated WT and *Ufm1*-KO BMDMs. **(F and G)** qPCR analysis of *Il1b* (F) and *Nlrp3* (G) mRNA expression in WT and *Ufl1*-KO BMDMs stimulated with LPS for indicated time periods. Data are representative of three independent experiments (A – E). Data are shown as mean ± SD (F and G). NS, no significance; *, P < 0.05. P values were determined by unpaired two-tailed Student’s *t* test.

Related to Figure 1.


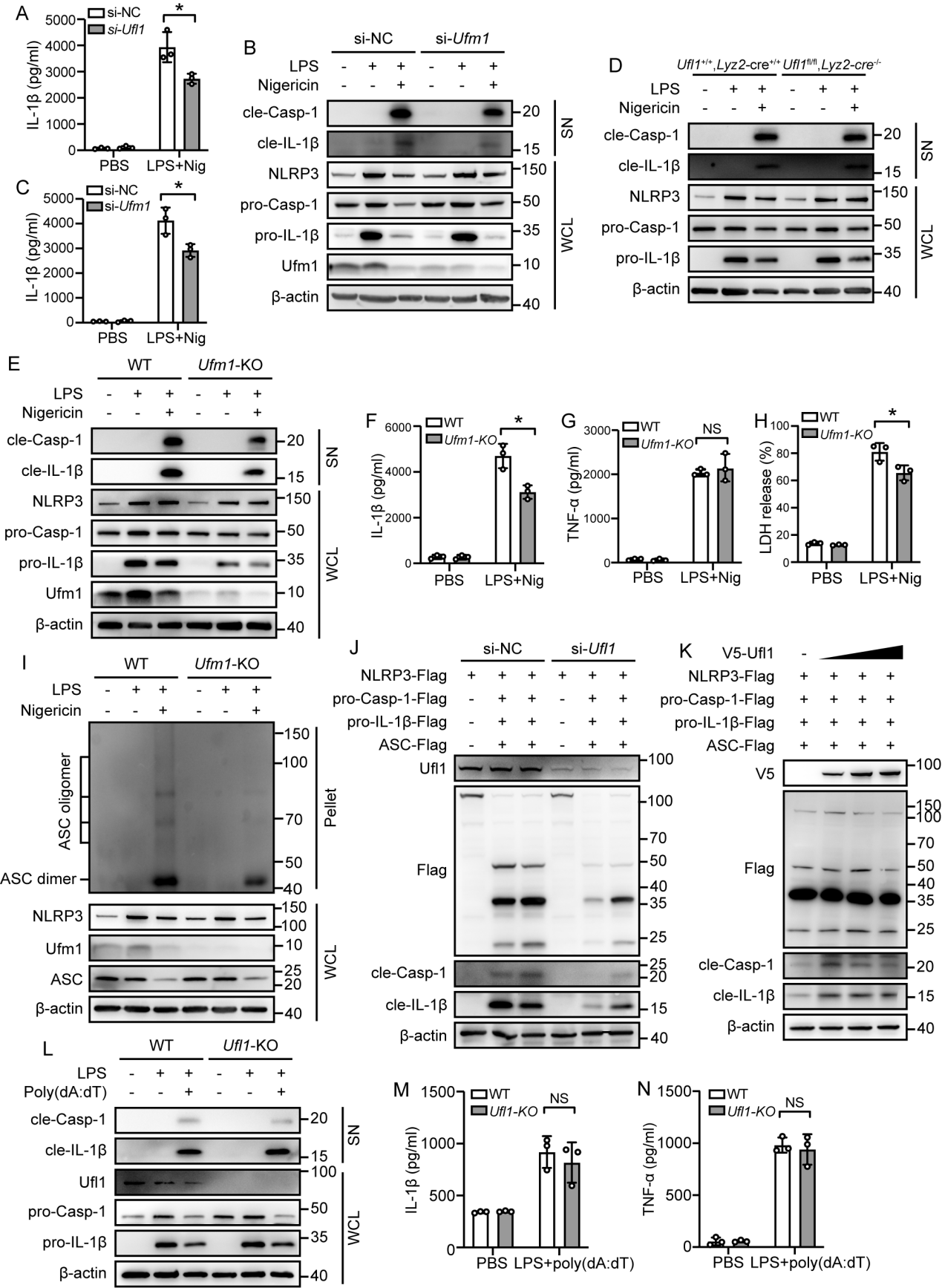


**Figure S2. Deficiency of *Ufm1* or *Ufl1* inhibits the activation of NLRP3 inflammasome. (A)** IL-1β production in SN of LPS-primed and nigericin-activated iBMDMs transfected with si-NC or si-*Ufl1*. **(B)** Immunoblot analysis of proteins in SN and WCL of LPS-stimulated, LPS-primed and nigericin-activated iBMDMs transfected with si-NC or si-*Ufm1*. **(C)** IL-1β production in SN of LPS-primed and nigericin-activated iBMDMs transfected with si-NC or si-*Ufm1*. **(D)** Immunoblot analysis of proteins in SN and WCL of LPS-stimulated, LPS-primed and nigericin-activated WT (*Ufl1*^+^/^+^, *Lyz2*-Cre^+/+^ and *Ufl1*^fl^/^fl^, *Lyz2*-Cre^-/-^) BMDMs. **(E)** Immunoblot analysis of proteins in SN and WCL of LPS-stimulated, LPS-primed and nigericin-activated WT and *Ufm1*-KO BMDMs. **(F - H)** IL-1β (F), TNF-α (G) production and LDH release (H) in SN of LPS-primed and nigericin-activated WT and *Ufm1*-KO BMDMs. **(I)** Immunoblot analysis of ASC oligomerization in pellets and WCL of LPS-primed and nigericin-activated WT and *Ufm1*-KO BMDMs. **(J)** Immunoblot analysis of Flag, cle-Casp-1, and cle-IL-1β of lysates from HEK293T cells transfected with si-NC or si-*UFL1*, and then transfected with indicated plasmids with different concentrations. **(K)** Immunoblot analysis of V5, Flag, cle-Casp-1, and cle-IL-1β of lysates from HEK293T cells transfected with indicated plasmids with different concentrations. **(L)** Immunoblot analysis of proteins in SN and WCL of LPS-stimulated, LPS-primed and poly(dA:dT)-activated (2 μg/ml, 6 h) WT and *Ufl1*-KO BMDMs. **(M and N)** IL-1β (M) and TNF-α (N) production in SN of LPS-primed and Poly(dA:dT)-activated WT and *Ufl1*-KO BMDMs. Data are representative of three independent experiments (B, D, E, I – L). Data are shown as mean ± SD (A, C, F – H, M, N). NS, no significance; *, P < 0.05. P values were determined by unpaired two-tailed Student’s *t* test.

Related to Figure 2


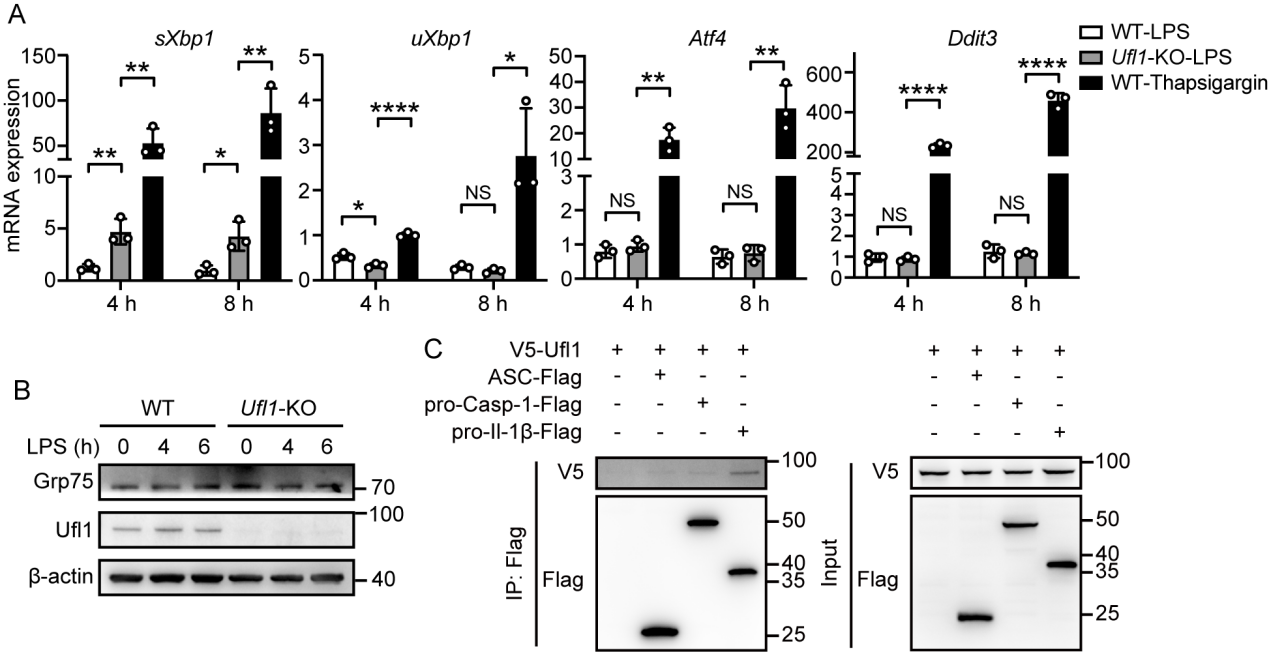


**Figure S3. *Ufl1* deficiency doesn’t induce ER stress response in LPS-simulated BMDMs, but solely the IRE1 branch. (A)** qPCR analysis of *sXbp1*, *uXbp1*, *Atf4*, and *Ddit3* mRNA expression in LPS-stimulated WT, *Ufl1*-KO BMDMs, and ER stress inducer (Thapsigargin) stimulated BMDMs at indicated time points. **(B)** Immunoblot analysis of Grp75 in lysates of WT and *Ufl1*-KO BMDMs stimulated with LPS for indicated time periods. **(C)** IP analysis of the association between Ufl1 and ASC, pro-Caspase1, or pro-IL-1β in HEK293T cells transfected with the indicated plasmids. Data are representative of three independent experiments (B and C). Data are shown as mean ± SD (A). NS, no significance; *, P < 0.05; **, P < 0.01; ****, P < 0.0001. P values were determined by unpaired two-tailed Student’s *t* test.


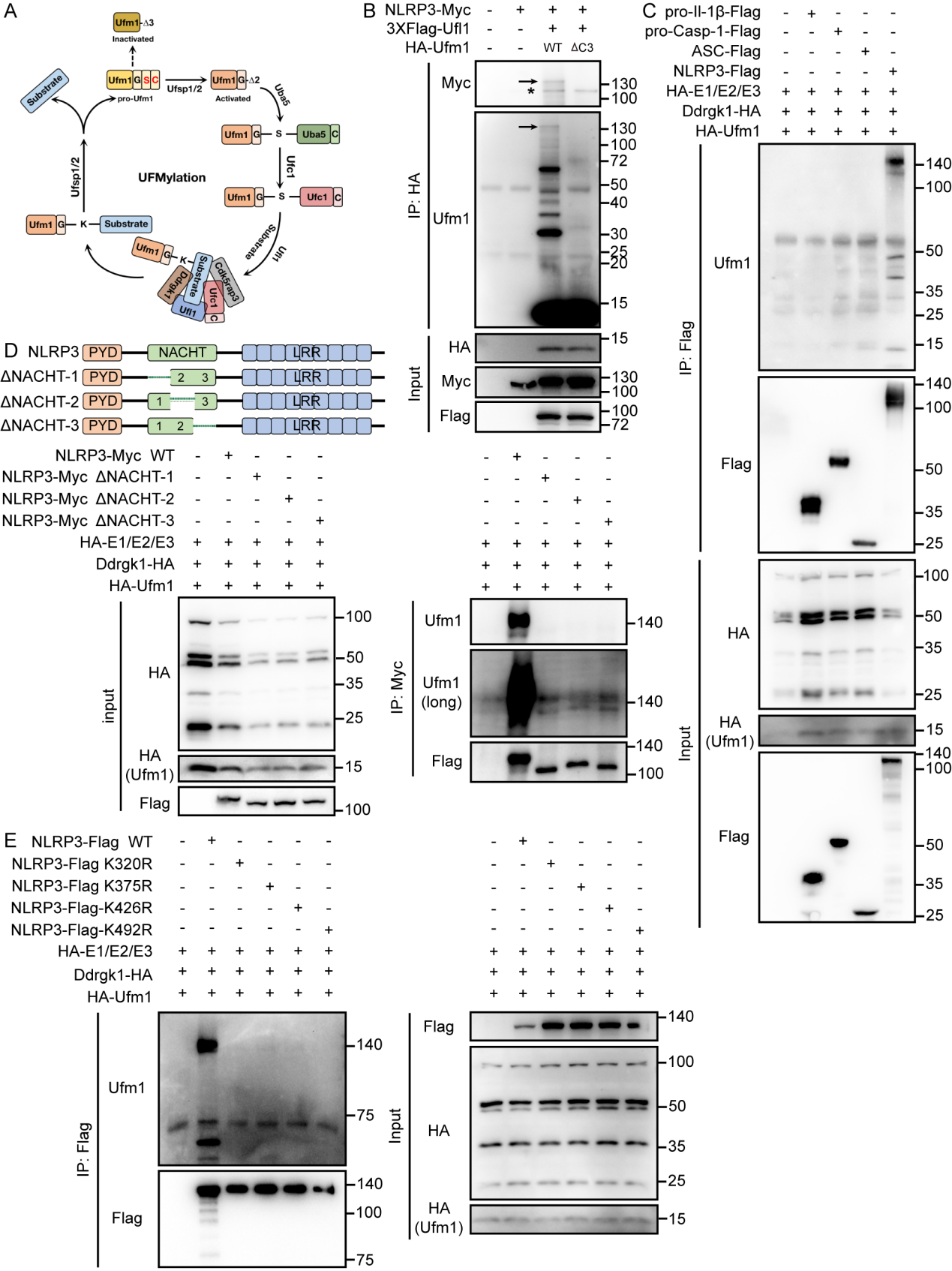


**Figure S4. UFMylation of NLRP3 truncation mutants is blocked. (A)** Schematic diagram of UFMylation modification process. **(B)** UFMylation of expressed NLRP3 was analyzed by the HA-IP in denaturing buffer A from HEK293T cells transfected with the indicated constructs. The arrow indicates NLRP3 band, the star represents the non-specific bind. **(C)** IP analysis of UFMylation of NLRP3 inflammasome components (pro-caspase-1, pro-IL-1β, and ASC) in HEK293T cells transfected with the indicated plasmids. **(D)** Schematic diagram of NLRP3 and its truncation mutants (NLRP3-∆NACHT-1, ∆NACHT-2, ∆NACHT-3) (top); IP analysis of UFMylation of NLRP3 or NLRP3 mutants in HEK293T cells transfected with the indicated plasmids (bottom). **(E)** IP analysis of UFMylation of NLRP3 or NLRP3 mutants (NLRP3 K320R, K375R, K426R, K492R) in HEK293T cells transfected with the indicated plasmids. Data are representative of three independent experiments.


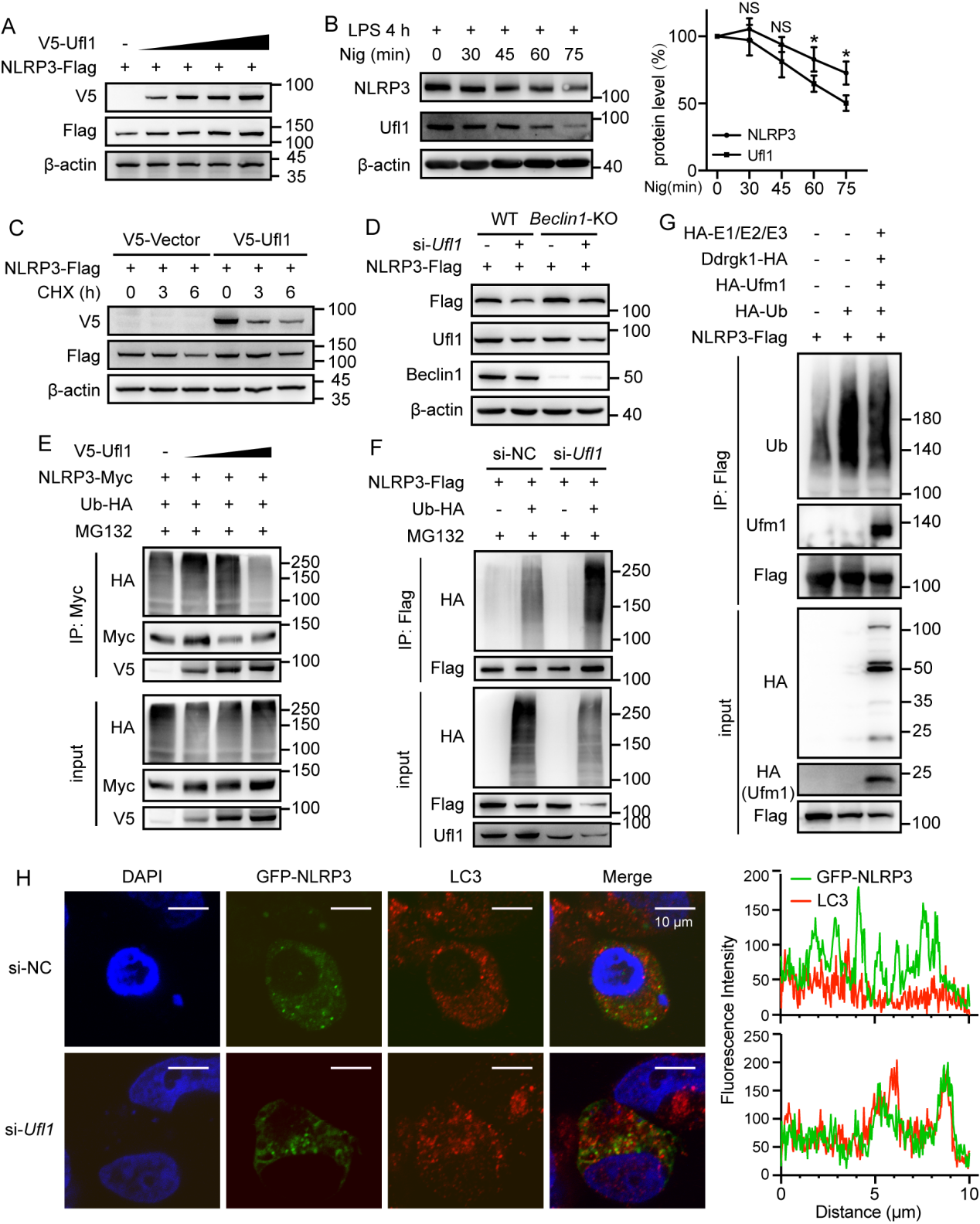


**Figure S5. *Ufl1*-mediated the degradation of NLRP3. (A)** Immunoblot analysis of NLRP3 expression in HEK293T cells transfected with indicated plasmids for different concentration. **(B)** Immunoblot analysis of NLRP3 and Ufl1 in lysates of WT stimulated with LPS and nigericin for indicated time periods (left). NLRP3 and Ufl1 expression was quantitated by measuring band intensities using ImageJ software (right). **(C)** Immunoblot analysis of NLRP3 expression in HEK293T cells transfected with indicated plasmids, following stimulation with LPS and then treated with CHX (100 ng/ml) for the indicated time periods. **(D)** Immunoblot analysis of NLRP3 expression in WT and *Beclin1*-KO HEK293T cells transfected with NLRP3-Flag and si-NC or si-*Ufl1*. **(E)** IP analysis of ubiquitination of NLRP3 in HEK293T cells transfected with the indicated plasmids, following treatment with MG132 (10 μM) for 8 h. **(F)** IP analysis of lysates from HEK293T cells transfected with si-NC or si-*UFL1,* Ub-HA, and NLRP3-Flag, following treatment with MG132 (10 μM) for 8 h and IP with Flag antibody. **(G)** IP analysis of ubiquitination of NLRP3 in HEK293T cells transfected with the indicated plasmids. **(H)** Immunofluorescence analysis of the association between NLRP3 (green) and LC3 (red) in HEK293T cells transfected with GFP-tagged NLRP3, following incubation with anti-LC3 and specific secondary antibody (left). Line scan graphs showing the immunofluorescence intensity using ImageJ software (right). Scale bar, 10 µm. Data are representative of three independent experiments. Data are shown as mean ± SD (B, right panel). NS, no significance; *, P < 0.05. P values were determined by unpaired two-tailed Student’s *t* test.


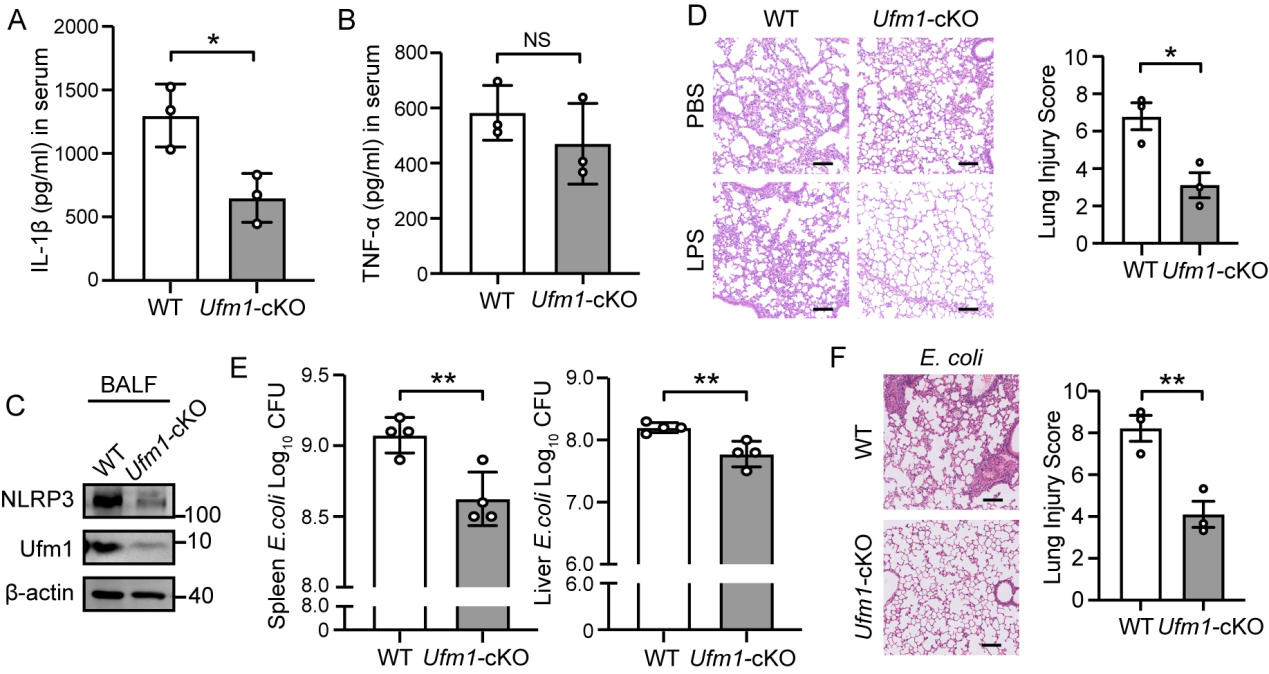


**Figure S6. *Ufm1* deficiency alleviates NLRP3 inflammasome activation *in vivo*. (A-D)** WT or *Ufm1-*cKO mice (n = 3 per group) were intraperitoneally (i.p.) injected with lipopolysaccharide (LPS, 15 mg/kg, 8 h). IL-1β (A) and TNF-α (B) release in serum was determined by ELISA. Immunoblot analysis of lysates from bronchoalveolar lavage fluids (BALFs) from WT or *Ufm1-*cKO mice (C). Representative images of H&E staining of lung tissues of WT and *Ufm1-*cKO mice. Scale bar, 100 µm (D). **(E and F)** WT or *Ufm1-*cKO mice (n = 4 per group) were injected with *E. coli* (1×10^9^/kg) for 24 h. Analysis of bacterial load in spleen (left) and liver (right) of WT and *Ufm1-*cKO mice (E). Representative images of H&E staining of lung tissues of WT and *Ufm1-*cKO mice. Scale bar, 100 µm (F). Data are representative of three independent experiments (C, D, and F). Data are shown as mean ± SD (A, B, D - F). NS: no significance; *, P < 0.05; **, P < 0.01. P values were determined by unpaired two tailed Student’s *t* test of n = 3 or 4 independent biological mice per group.
